# Tuning the Dimensionality of Protein–Peptide Coassemblies to Build 2D Conductive Nanomaterials

**DOI:** 10.1021/acsnano.4c18613

**Published:** 2025-04-25

**Authors:** Laura Perez-Chirinos, Lisa Almonte, Juan David Cortés-Ossa, Eduardo Solano, M. Reyes Calvo, Ivan R. Sasselli, Aitziber L. Cortajarena

**Affiliations:** † Center for Cooperative Research in Biomaterials (CIC biomaGUNE), Basque Research and Technology Alliance (BRTA), Paseo de Miramón 194, Donostia-San Sebastián 20014, Spain; ‡ Instituto Universitario de Materiales de Alicante (IUMA), Universidad de Alicante, Alicante 03690, Spain; § BCMaterials, Basque Center for Materials, Applications and Nanostructures, UPV/EHU Science Park, Leioa, Vizcaya 48940, Spain; ∥ NCD-SWEET Beamline, ALBA Synchrotron Light Source, Cerdanyola del Vallès, Barcelona 08290, Spain; ⊥ Centro de Física de Materiales (CFM), CSIC-UPV/EHU, Paseo Manuel de Lardizabal 5, Donostia-San Sebastián 20018, Spain; # IKERBASQUE, Basque Foundation for Science, Plaza Euskadi 5, Bilbao 48009, Spain

**Keywords:** peptide design, protein engineering, self-assembly, peptide−protein coassemblies, supramolecular fibers, protein paracrystals, conductive materials

## Abstract

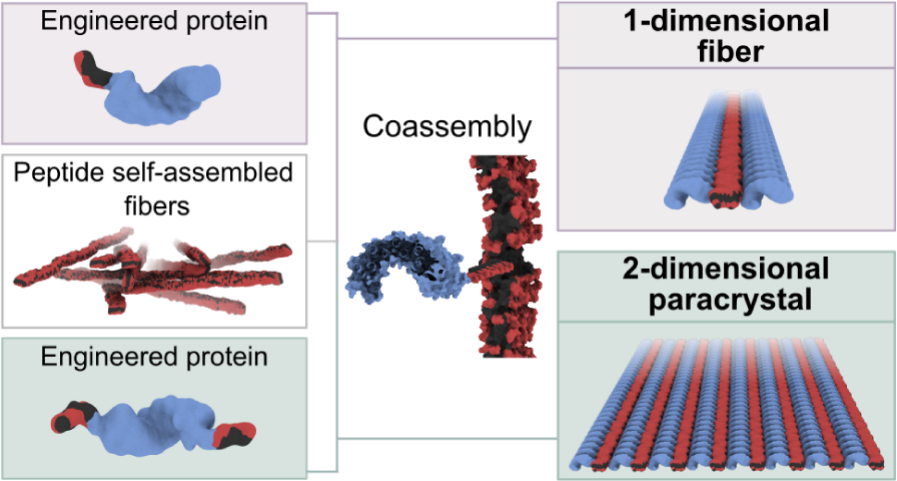

The natural self-assembly tendency of proteins to build complex structural architectures has kindled inspiration in developing supramolecular structures through the rational design of biomacromolecules. While there has been significant progress in achieving precise control over the morphology of self-assembled structures, combining different molecules within assemblies enables the design of materials with increased complexity, sophisticated structures, and a broad spectrum of functionalities. Here, the development of 1D and 2D peptide–protein coassembled systems based on the design of amphiphilic peptides and engineered proteins is described. The peptide was optimized to form stable self-assembled fibers by evaluating, computationally and experimentally, the assembling tendencies and the supramolecular features of peptides with different lengths and negative charges. A superhelical repeat protein was engineered by fusing one or two amphiphilic peptides into one or both termini. This modification drove the coassembly between the self-assembled fibers and the protein with one or two peptides, resulting in 1D or 2D coassembled systems. The protein films and the 2D coassembled system exhibited high ionic conductivity for a biomolecular system, attributed to their high content of charged residues, positioning these materials as promising candidates for developing bioelectronic devices. Thus, this work provides a versatile framework for developing coassembled materials with tunable dimensionality by using biocompatible building blocks without any additional chemical moieties, highlighting the potential for their use in biocompatible electronics.

The inherent ability of nature to form ordered structures from scratch has inspired scientists to design complex materials using biomolecular building blocks for numerous applications. Proteins are one example of molecules capable of generating supramolecular ordered architectures through spontaneous self-assembly of the individual components.^1−11^ This process has enabled the rational design of peptides and proteins to construct highly specialized functional materials.^12−21^ The advances in understanding and controlling the development of these materials based on the design of biomolecules have led to significant innovations in cell culture, tissue engineering, and bioelectronics, among others.^22−27^

Toxic materials used in electronic devices pose environmental risks and are unsuitable for biomedical use,^28^ prompting interest in biodegradable and biocompatible alternative materials as platform for biomedical electronic devices for monitoring, diagnostics, and therapeutic interventions.^29−33^ To this end, the electrical properties of biomolecular assemblies have been explored, demonstrating their potential as robust candidates for the development of bioelectronic materials.^34−37^ For instance, self-assembled peptide fibers have previously presented electronic conductivity due to electronic delocalization from the π-π interactions that drive their assembly.^38−42^ Additionally, the conductivity of protein-based materials can be enhanced in hybrid systems by combining proteins with other electroactive materials such as nanoparticles or conductive polymers.^24,43,44^ Moreover, different peptide assemblies and protein-based materials have exhibited efficient ionic conduction properties,^24,45−49^ particularly through proton hopping mechanisms, making them good ionic conductors in humid environments. This intersection between molecular electronics and biology is a challenging yet promising research area with great potential for developing biocompatible and sustainable conductive materials, enabling us to deepen our understanding of bioelectrical mechanisms in natural systems.

Amphiphilic peptides can spontaneously form supramolecular structures in aqueous solution by concealing the hydrophobic component within the core and exposing the hydrophilic motifs to the surface, stabilizing the interphase with water. The properties of the resulting material are encoded in the amino acid sequence, and even small modifications in sequence hydrophobicity,^50,51^ charge density,^52^ chirality of the amino acids,^53,54^*etc.*, can strongly affect the supramolecular morphology.^55,56^ This enables precise control through rational design^14−20^ over some of their features, such as intermolecular cohesion, assembly tendencies, and charge distribution.^57^ The optimization of these features enables the development of supramolecular structures with tunable and precise properties and functions, demonstrating excellent performance in various applications, including exceptional bioactive properties when employed as artificial extracellular matrices^22^ and conductive properties for their use in bioelectronics.^52^ However, the sequence/structure correlation is not fully understood and the effect of this variation is often difficult to predict. Consequently, computational methods have recently emerged as a complementary approach to better understand how these changes tune the structures at the intermolecular level, allowing to narrow down the vast number of possible peptide sequences and effectively screening and predicting promising candidates for the development of supramolecular materials.^58−62^

Proteins offer a natural versatility for developing materials with targeted functionality and properties through the design of their sequence.^63^ Some proteins present inherent self-assembling tendencies, forming highly ordered structures such as keratin, collagen, S-layers, or viral capsids.^1−3,16^ Inspired by these natural tendencies, the rational design of proteins has enabled the development of protein-based materials via optimized hydrophobic interfaces,^18^ biotin–streptavidin linkage,^64^ disulfide bonds,^65^ peptide-assisted linking,^66^ and metal-assisted interactions.^65^ However, the complexity of these biomolecules makes it challenging to fully control the effects of mutations on their structural integrity. Computational methods have also played a pivotal role in addressing these challenges by contributing to understanding the relationship between protein sequence and structure, enabling novel protein design.^67^ Simplified protein systems, such as repeat proteins, have emerged as valuable choices for overcoming the complexity of natural biomolecules, given their tunable size and morphology, which make them ideal for engineering robust protein scaffolds.^68^ Their inherent modularity makes them ideal candidates for developing self-assembled materials.^68,69^ In particular, consensus tetratricopeptide repeat (CTPR) proteins consist of a helix-turn-helix structural building block of 34 amino acids. Extended arrays of multiple repeats fold into a right-handed superhelix.^70−73^ Their modularity drives innate self-assembling properties via “head-to-tail” and “side-to-side” interactions, as observed in their crystal structures,^72,74^ enabling the development of CTPR-based materials with controlled properties.^20,75−77^

The combination of different building blocks can extend the properties and applications of these materials without introducing significant complexity to the design.^12^ Coassemblies between designed peptide molecules have been widely studied as they allow tuning the physical properties as well as introducing functional components to the systems, achieving outstanding results in tissue engineering and biomedicine.^12,78,79^ Similarly, the development of materials based on the combination of different protein building blocks has also been employed to gain further control over supramolecular structures.^18^ However, the combination of peptides and proteins in supramolecular structures has been sparsely explored. Heterogeneous membranes that act as bioactive scaffolds have been recently studied in literature, which can be obtained from the aggregation between peptides and proteins.^80^ However, the development of materials which involve the combination of peptides and proteins are mostly built by unspecific interaction between the components, focusing mainly on the incorporation of proteins as functional units rather than as a structural component.^81^ Although synergistically exploiting the structural capabilities of proteins and peptides in coassemblies remains a formidable challenge due to the complexity of integrating both components, it holds great promise due to the structural and functional capabilities of each component and the advantages their combination can offer. This has been previously explored by Hudalla *et al.*, where they developed ordered peptide–protein coassembled fibers with fluorescence and enzymatic capabilities.^82^ Moreover, the rigidity, bulkiness, and robust structure provided by proteins play a crucial role in the formation of ordered coassemblies by preventing fibers from self-interacting—a phenomenon more likely to occur with small molecules such as peptides or DNA—while also minimizing bending and undesired interactions between the fibers.^79^

In this work, the development of protein–peptide coassemblies by leveraging the structural properties of both components to control the dimensionality of the resulting nanostructured biomaterials is presented. To achieve this, peptides consisting of phenylalanine and glutamic acid repeats (FE)_*n*_ were used. First, different lengths (*n*) of repetitions of the amphiphilic dipeptide were screened to obtain the optimal sequence for peptide self-assembly. Further optimization was conducted by evaluating the impact of additional negative charges at the peptide termini on their supramolecular properties. Next, the design of engineered CTPR proteins was performed to obtain scaffolds suitable for the development of coassembled 1D fibers and 2D paracrystals. This was achieved by fusing the optimized peptide sequence to one or both termini of a CTPR8 protein, *i.e*., a CTPR protein with 8 identical CTPR repeats, resulting in the recombinant expression of the final protein–peptide construct. Finally, the resulting 2D coassembly was employed to develop conductive systems, leveraging its intrinsic biocompatibility, precise spatial organization, and incipient conductive properties. These features underscore its potential application in bioelectronics and related fields.

## Results and Discussion

### Optimization of a Peptidic Sequence for the Development of Supramolecular Self-Assembled Fibers

**Figure 1:**
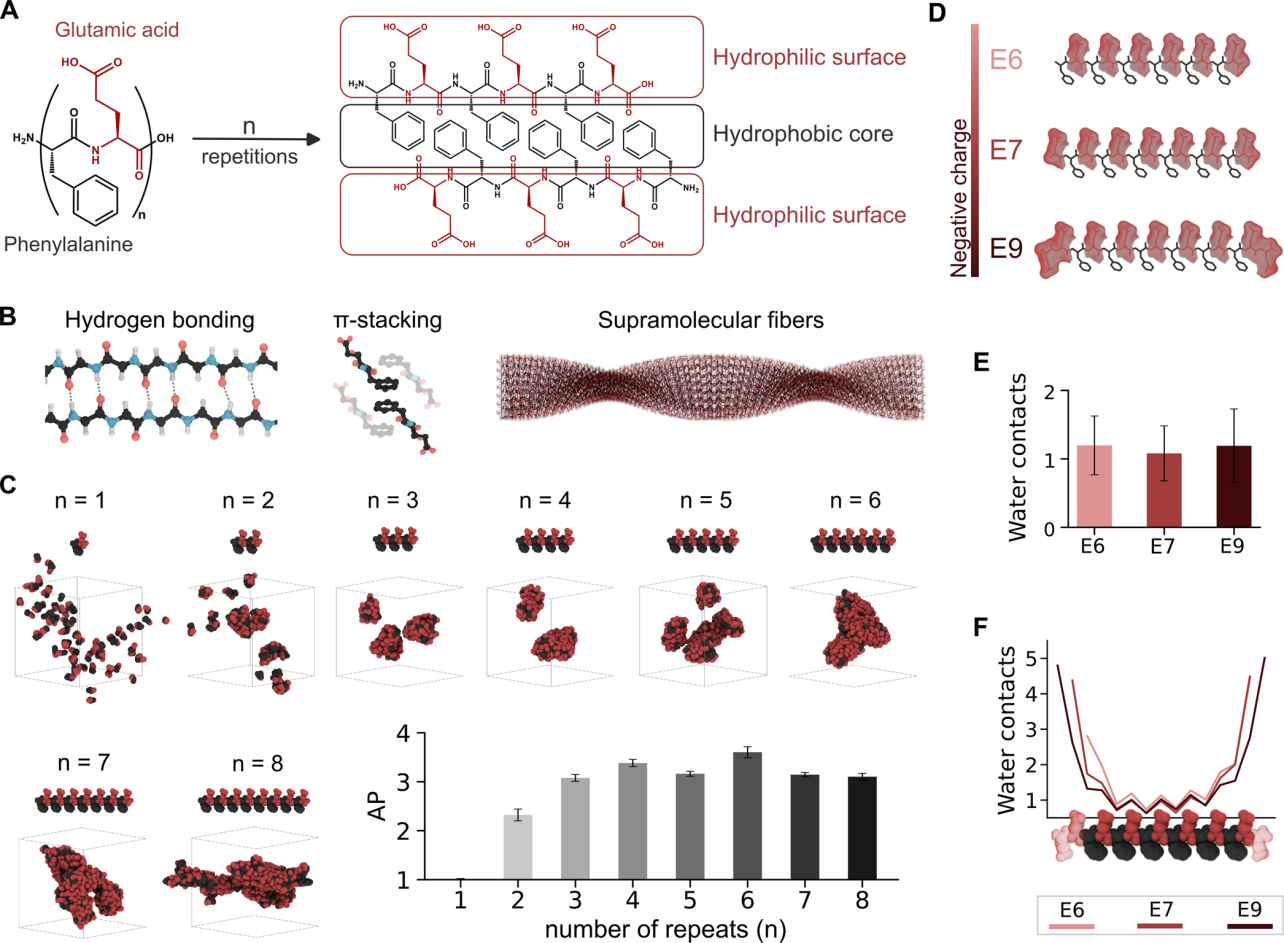
Peptide design. A. Structural representation of the dipeptide and the assembly of peptides with *n* repetitions. B. Interactions involved in the stabilization of the supramolecular fibers: Hydrogen bonding (left), π-stacking (middle), and a theoretical model of the supramolecular fiber (right). C. Snapshots of the last frame of the CG-MD simulations and AP of the peptides from 1 to 8 repetitions of the (FE)_*n*_ dipeptide. D. Schematic representation of the designed peptides. **E6** with 6 net negative charges, **E7** with 7, and **E9** with 9. E. Averaged water contacts (number of water beads in contact with each backbone bead) for the last 0.5 microsecond of the CG-MD simulations, excluding the N- and C-terminus beads. F. Water contacts of each backbone bead as represented in the structure at the bottom. The transparent glutamic acids illustrate the extra negative residues in the **E7** and **E9** structures, being the common residues between the three designs (**E6**, **E7**, and **E9**) opaque.

The designed sequences were based on the repetition of a dipeptide composed of one phenylalanine (F) and one glutamic acid (E). The phenylalanine residues are critical to driving the aggregation as the initial step of self-assembly due to their hydrophobicity while also contributing with π-stacking interactions to the final order of the assembly. The glutamic acid residues are hydrophilic and thus stabilize the interphase of the structures with water. Alternating the glutamic acid and phenylalanine residues results in an amphiphilic molecule with a hydrophobic and a hydrophilic side (Figure 1A). These peptides were designed to spontaneously self-assemble via the aforementioned hydrophobic interactions and π-stacking of the phenylalanine side chains aided by the hydrogen bonds between the backbone of the peptides in one-dimensional (1D) structures (Figure 1B), inspired by previous examples.^50,51^ The resulting structures have a phenylalanine-rich hydrophobic core and a glutamic-rich surface that increases the solubility of the fiber and prevents the bundling through charge repulsion between the fibers. Most importantly, these negative charge densities avoid nonspecific interactions with the also negatively charged surface of CTPR proteins, which is crucial to ensuring the correct coassembly between the peptides and the proteins.

We hypothesized that the number of FE repeats would play a significant role in the ability of the resulting peptide to self-assemble. Therefore, the effect of the peptide length (*n*) in (FE)_*n*_ peptides to form supramolecular structures was studied through coarse-grained molecular dynamics (CG-MD) simulations by inserting randomly the peptides in a simulation box and evaluating their tendency to aggregate.^83^ Relative self-assembling trends in this series were assessed employing the aggregation propensity (AP) of the peptides by calculating changes in the solvent-accessible surface area (SASA) as described by Frederix et al.^58^ This parameter represents the inverse relative solvent exposure at a given time. An AP value of 1 corresponds to totally soluble peptides, while AP > 2 represents a high likelihood of peptide self-assembly. The AP parameter has been reported in the literature as an indicator of the tendency of a peptide to experimentally form self-assembled fibers. As the SASA of the peptides decreases upon aggregation, a higher AP value indicates a greater tendency to form such fibers.^58,59,84^ The results showed that from *n* = 2 the peptides start to aggregate, observed by an AP value >1 and by the snapshots. Increasing the number of repeats enhances the AP, reaching a maximum of aggregation at *n* = 6. Meanwhile, even though beyond six repeats of the dipeptide the snapshots show an aggregate similar to the six-repeat peptide, their AP value decreases (Figures 1C, S1A). This may be due to the increase in the number of glutamic acids enhancing solubility or affecting the peptide’s self-conformation competing with self-assembly. Therefore, the 6-repeat peptide (FEFEFEFEFEFE), now called **E6**, was chosen for its optimal aggregation properties.

The selected **E6** sequence contains one phenylalanine at the N-terminus, which would lie relatively exposed to the solvent at the fiber edge. Considering that the exposure of this hydrophobic residue on the assembly surface could cause destabilization or fiber branching, additional negative charges were added to one or both termini of the peptides to analyze their effect on the supramolecular structures. The two new peptide sequences are EFEFEFEFEFEFE (**E7**) and EEFEFEFEFEFEFEE (**E9**) (Figure 1D). We employed CG-MD simulations to assess the effect of these additional charges in their self-assembly and analyzed the water contacts along the peptide sequence to gain further details on peptide disposition (Figures 1E,F, S1B). The results showed lower water contacts in the center of the sequence, in good agreement with the expected preferential stack of the peptides (Figure 1A). The decrease in the number of contacts with water molecules of the backbone beads of the phenylalanines in comparison with the backbone beads of the glutamic acids suggests that the charged residues are exposed to the solvent whereas the aromatic residues are hidden from the water (Figures 1F, S1B). This supports that the core of the fibers is formed by the side chains of the phenylalanines, driven by hydrophobic interactions and facilitating their π-stacking, with the charged side chains of the glutamic acids disposed on their surface, as expected from the design strategy. The increment in charge did not significantly affect their self-assembly presenting only minimal differences in their contacts with water in the central residues. Moreover, the lower averaged water contacts of the backbone beads of **E7**, excluding the N- and C-terminus beads (Figure 1E), could indicate, based on previous works,^85^ a higher intermolecular order of this sequence. This effect arises from the ability to form hydrogen bonds being proportional to the system’s capacity to exclude water molecules that could compete with these interactions.

**Figure 2:**
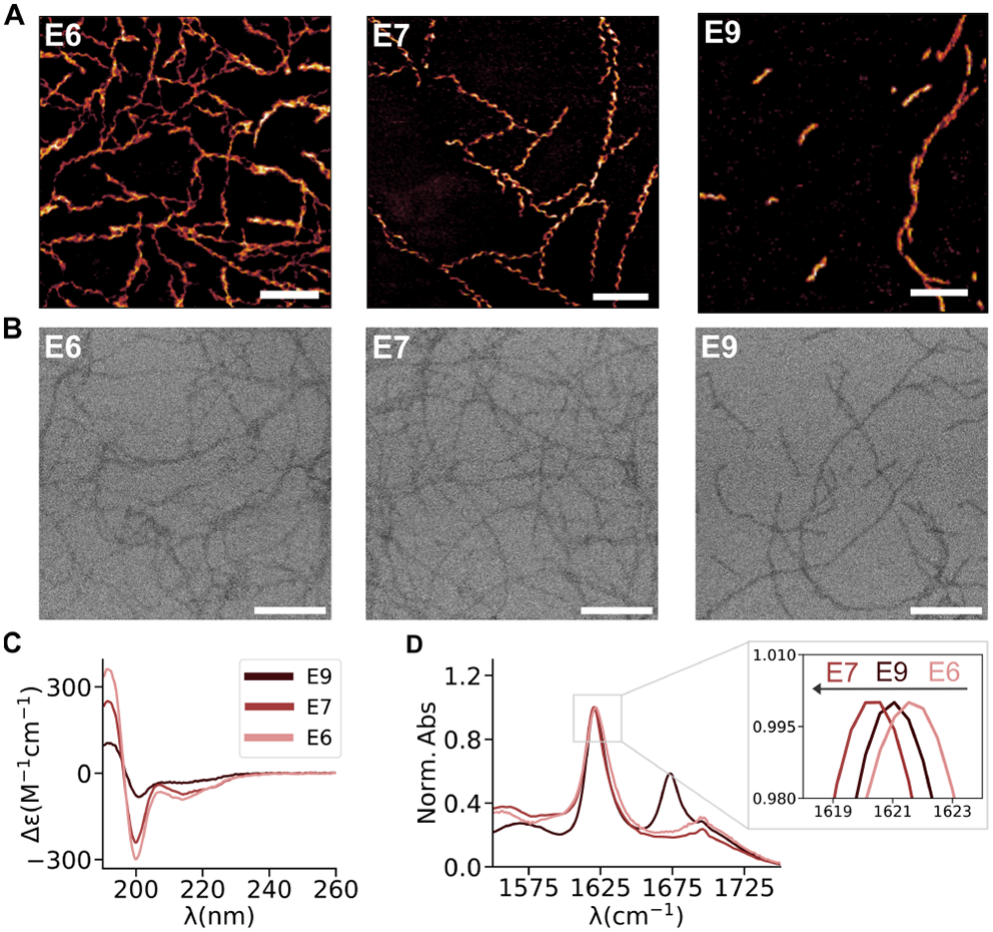
A. AFM topography and B. TEM images of the corresponding E6, E7, and E9 peptide self-assembled fibers. C. CD spectra of the fibers. D. FT-IR spectra of the fibers. All scale bars represent 100 nm.

Experimentally, the ability of all three peptides to self-assemble into 1D structures was studied through AFM and TEM at pH ≈ 5, which was required to obtain fibers. At higher pHs no self-assembly was observed, most likely due to the strong repulsion among negative charges of totally deprotonated glutamic acid side chains, which keep the peptide in their soluble form. Notably, pH 5 seems to be close enough to the p*K*_a_ of these side chains (4.25) to achieve neutralization of a fraction of these acids, reducing the repulsion and triggering the hydrophobic-driven aggregation of the peptides as first step of the self-assembly. Interestingly, CG-MD simulations did not require such charge neutralization, probably because the repulsion was overcome by the overestimated hydrophobic interactions in Martini 2.2.^66^ Consistent with these predictions, the results confirm that the three peptides form supramolecular fibers (Figure 2A,B, S2A) of a similar width of around 5 nm (Figure S2B). Regarding the length of the fibers, **E6** and **E7** did not show significant differences (Figure 2A). However, it is possible to appreciate on the left side of the AFM image of **E9** a high number of fibers that are clearly shorter than those of **E6** and **E7** (Figure 2A). The intermolecular order in the fibers was analyzed in terms of the formation of secondary structures through circular dichroism (CD) and Fourier-transformed infrared (FT-IR) spectroscopy in solution. The CD (Figure 2C) spectra showed a positive peak at 195 nm and two negative peaks, one at 200 nm and another at 215–230 nm, for the three peptides. The positive peak at 195 and the negative peaks in the region of 215–230 nm are characteristic of ß-sheet formation in peptides and proteins, in line with the proposed intermolecular arrangement for these peptides. The second peak around 200 nm is not common in protein structures, but it has been previously observed in assemblies of short peptides with a high number of aromatic side chains, arising from the contribution of extended π-stacking interactions.^86^ The peaks of **E9** are significantly less intense, suggesting that the additional charges may destabilize the assembly, in line with the limited length of the fibers formed by this peptide observed by TEM and AFM (Figure 2A,B). Instead, **E7** and **E6** showed stronger signals in both contributions. Regarding the FT-IR, the three peptides displayed peaks in the region 1615–1625 cm^–1^, typical of ß-sheet order (Figure 2D). As the shift to lower wavenumbers is related to the increment of hydrogen bonding order, **E7** presented the highest order, followed by **E9**, and **E6** showed the lowest fiber stabilization. The differences observed in **E9** between CD and FT-IR could be attributed to the additional negative charges, which, while not favoring well-ordered secondary structures, may promote a certain level of hydrogen bonding enhancement. However, interestingly, **E7** presented the strongest hydrogen bonding (Figure 2D), which correlates with the lowest number of water contacts of the backbone packing (Figure 1E). Therefore, both FT-IR and the simulations reveal **E7** as the peptide with the highest indicators of intermolecular order, only subtly surpassed by **E6** in the CD measurement but showing the lowest hydrogen bonding strength in the FT-IR. Additionally, the potential exposure of phenylalanine residues in the **E6** structures may enhance unspecific hydrophobic interactions among fibers and with the proteins, which could potentially disrupt the uniform coassembly. Therefore, **E7** was selected as the candidate for coassembly with the proteins.

### Design of Engineered Protein–Peptide Complexes

**Figure 3:**
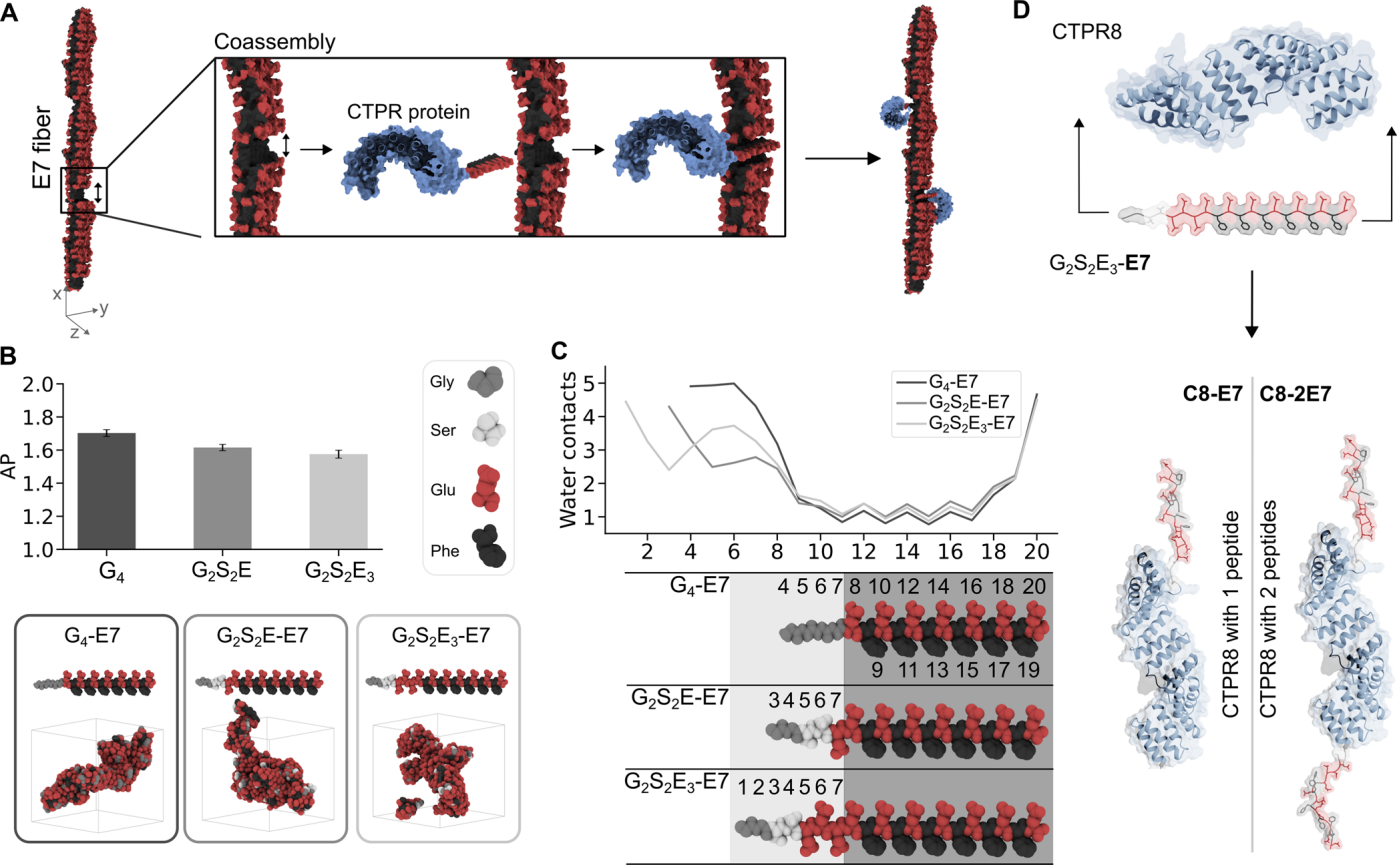
Protein design. A. Strategy of the coassembly. B and C. Computational screening of the linker sequence. B. Average of the AP only of the linker sequence during the last 0.5 microsecond (top). Snapshot of the last frame of the CG simulation of the peptides with the linkers (bottom). C. Water contacts of each backbone bead of the whole sequence (top). Numbers of the beads for each peptide in the water map (bottom). The peptidic sequence (dark gray) has the same numbering for all the peptides, and the linker sequence (light gray) is the variable part with different numbering. D. Protein design. **E7** peptide with the linker (G_2_S_2_E_3_-E7) added to one or both termini of a CTPR8 obtaining the **C8-E7** and **C8-2E7** proteins, respectively.

The coassembly between the supramolecular fibers and the CTPR proteins was driven through the incorporation of the **E7** peptides to the protein termini, which could interlock into the supramolecular **E7** stack (Figure 3A). A CTPR with 8 repeats (CTPR8) was chosen for the development of the material as 8 repeats complete a superhelical turn.^72^ This is essential for controlling the planarity of the supramolecular structures in the *z*-dimension (Figure 3A), as the N-terminus of the protein aligns along the *y*-axis with the C-terminus, avoiding nondesired twists in the material which could hinder material growth. Thus, the CTPR8 protein was expressed with one or two **E7** peptides to form **C8-E7** and **C8-2E7**, respectively. **C8-E7** presents the **E7** peptide at the C-terminus to drive its incorporation into one **E7** fiber and form 1D coassemblies. Whereas the **C8-2E7** presents two **E7** peptides at both termini, promoting their incorporation into two different **E7** self-assembled fibers promoting the formation of a 2D coassembled system. The rigidity of the CTPR8 protein superhelix^87^ prevents both peptides from interacting with the same fiber, facilitating the role of **C8-2E7** as a cross-linker between different fibers.

**Figure 4:**
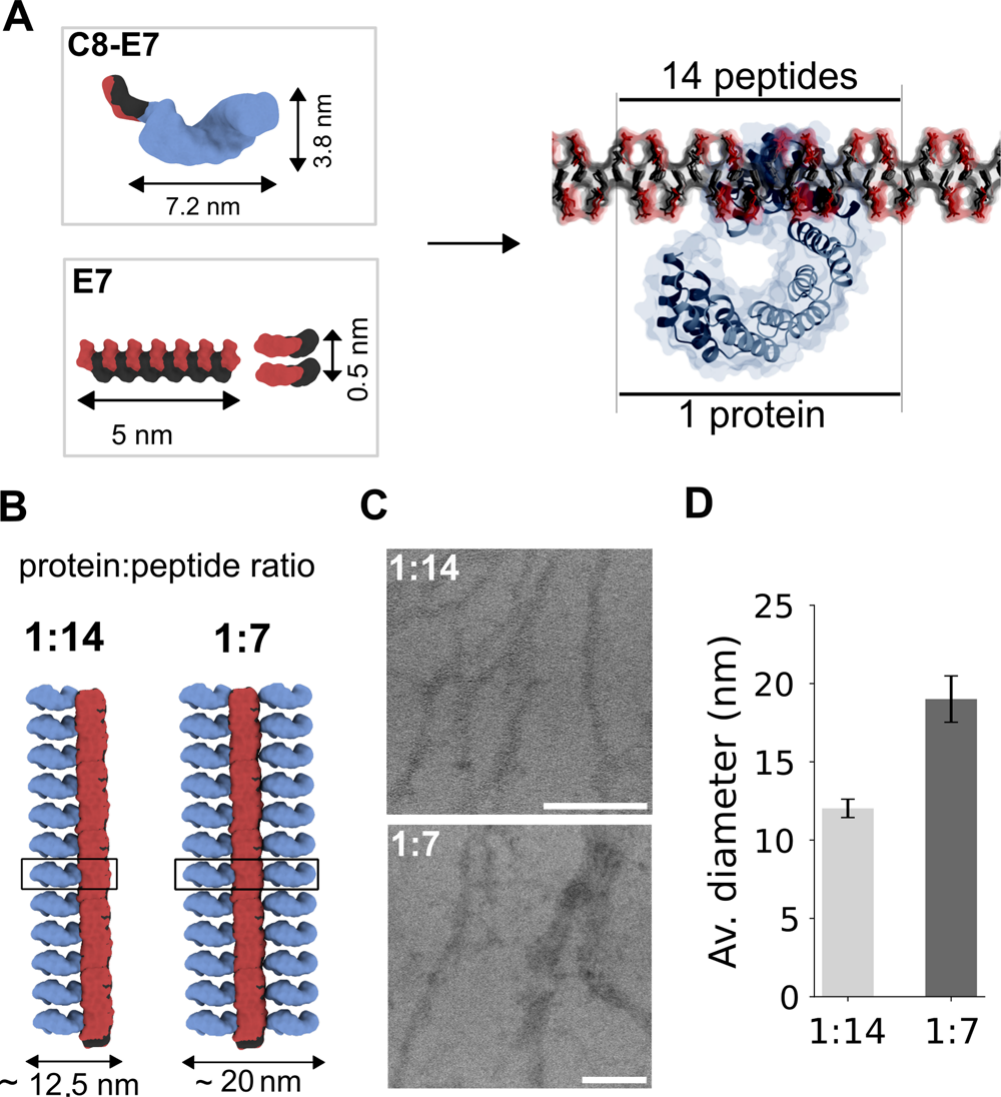
Design and characterization of the 1D coassembled fibers. A. Dimensions of the individual components and theoretical ratio and design of the 1D coassembled system. B. Graphical representation of the coassembly with a 1:14 and 1:7 protein:peptide ratios. C. TEM images of the 1D coassembled fibers with the 1:14 and the 1:7 protein:peptide ratios. Scale bars represent 100 nm. D. Averaged diameters from the TEM images at both ratios.

To facilitate the coassembly of the engineered proteins with the **E7** fibers, a linker was incorporated between the protein and the peptide. This linker was designed to increase flexibility and distance between the protein and the fibers to reduce the steric hindrance that could potentially disrupt the supramolecular order of the fibers. Initially, a peptide sequence of four glycines (G_4_-) was designed. Glycine was the residue chosen because of its lack of a side chain to impede steric hindrance. However, it was observed through CG-MD simulations that the AP of the G_4_- linker sequence was higher than expected for a soluble and flexible linker (Figure 3B). Therefore, to improve its solubility, the last two glycines were replaced with serines (S), and one (G_2_S_2_E-) or three (G_2_S_2_E_3_-) additional glutamic acids were also added to increase electrostatic repulsion between the **E7** peptide and the CTPR8. The lower AP of the G_2_S_2_E- and G_2_S_2_E_3_- linker sequences showed that the addition of these serines and glutamic acids enhances the solubility of the linkers (Figure 3B). Additionally, these linkers did not negatively affect the overall self-assembly of the peptide as observed through the similar number of contacts with the water of the **E7** peptidic sequence in all cases (Figure 3C). The G_2_S_2_E_3_- linker, from now on **L-E7**, was chosen for its higher solubility. To further test whether the linker at different concentrations might affect the assembly of the peptides, CG-MD simulations at different ratios of **E7** and **L-E7** peptides were performed (Figure S3). The **L-E7:E7** ratios for the coassembled systems were calculated based on the dimensions of the peptide and the protein. The dimensions of the CTPR8 superhelix are 3.8 nm wide and 7.2 nm long, as calculated from its crystal structure (PDB ID: 2AVP).^72^ The experimental width obtained for the **E7** fibers by AFM and TEM was ∼5 nm (Figure S6), in agreement with the theoretical length of a single peptide (4.94 nm for **E7**) considering that each amino acid contributes ∼0.38 nm to the peptide chain.^88^ Regarding the dimensions along the fiber, the distance between peptides within the fiber was estimated to be 0.5 nm given the distance between the peptide–peptide intermolecular interactions and the width of the peptide chain (Figure 4A).^89^ Given these dimensions and considering that two peptides faced each other to protect the hydrophobic core displaying two hydrophilic surfaces, the protein:peptide ratios calculated were 1:14 (eq S2) for a system with just one side of the fiber covered with proteins and 1:7 (eq S3) for a system with both sides of the fiber covered (Figure 4B). Additional ratios, 1:3 and 1:30, represent the excess of **L-E7** and **E7**, respectively (Figure S3A). In these simulations, the number of water beads in contact with the backbone beads of each residue, *i.e.*, the exposure of each residue to the solvent, was analyzed. These water maps showed that the residues that composed the linker part of **L-E7** had higher contact with water molecules than the amphiphilic part of **E7** and the **E7** “naked” peptides regardless of the quantity of **L-E7** (Figure S3B). This, along with the similarity in the water map profile between the **E7** with and without the linker, suggests that the modification had minimal impact on the self-assembly of the attached peptide. Instead, the linker appeared to position itself on the outer part of the fibers, exposed to the solvent. Therefore, given the lower AP of the G_2_S_2_E_3_- linker and the fact that this linker did not bury within the aggregates, aggregate itself, or affect the aggregation of the **E7** attached to it, this linker sequence was chosen to bind the **E7** peptide to the CTPR8 (Figure 3D).

The structural integrity of the engineered proteins was assessed both computationally, through all-atom molecular dynamics (AA-MD) simulations, and experimentally, through CD, to evaluate any potential disruptions in the secondary and tertiary structures of the CTPR caused by the addition of the amphiphilic peptide **E7**. The simulations presented low RMSD values averaging 0.33 nm, which indicates that the superhelical structure was not disrupted (Figure S4A). To experimentally assess the stability of both proteins, they were expressed and purified individually (Figure S4B). The purified proteins showed the expected increase in mass corresponding to the additional peptidic sequences (Figure S4C). Additionally, both CDs showed identical spectra characteristic of the TPR fold (Figure S4D), confirming that both proteins conserve their α-helical secondary structure. Together, these results demonstrate that the modified CTPR proteins maintain their characteristic structure after the addition of one or two **E7** peptides.

Even though the superhelical fold of the CTPR is conserved, the high content of phenylalanine residues in the amphiphilic peptide could trigger unspecific aggregation. This was studied experimentally through dynamic light scattering experiments (DLS). The results confirmed for both engineered proteins, **C8-E7** and **C8-2E7**, the presence of a monodispersed peak with a hydrodynamic radius (*R*_H_) of around 3 nm. This *R*_H_ value is slightly higher than the hydrodynamic radius measured for a CTPR8 (∼2.3 nm), which may be due to the addition of the **E7** peptides in the engineered proteins (Figure S4E). These results demonstrate that the additional hydrophobic residues in the **E7** peptides do not promote aggregation of the CTPR8, further supporting the suitability of the engineered **C8-E7** and **C8-2E7** for coassembly with the **E7** supramolecular fibers.

### Principle of the Coassembly

The coassembly combines two structural components: the **E7** self-assembled fibers and a CTPR8-engineered protein. The modified protein in the 1D coassembled fibers is **C8-E7**, whereas in the 2D coassembled system, the modified protein is **C8-2E7**. It is noteworthy that, although an annealing process to favor intermolecular order and formation of longer fibers is employed,^90^ peptides spontaneously form fibers immediately upon being dissolved in solution at the appropriate pH. In this context, to facilitate coassembly, the **E7** self-assembled fibers were partially disrupted by sonication and temperature, weakening the intermolecular forces among the peptides and thus creating space for the inclusion of the peptide attached to the engineered proteins. While the **E7** self-assembled fibers are relatively stable, the π-stacking is progressively weakened from 50 °C up to 90 °C (Figure S5A). Previous reports have shown a correlation between weaker intermolecular interactions and shorter fibers and, thus, we expect that increasing temperature leads to fiber shortening.^90^ Both the weakening of intermolecular interactions and fiber shortening contribute to creating the necessary interaction points for the **E7** peptide fused to the CTPR protein to be incorporated into fiber. Additionally, it must be taken into account that proteins must remain folded during the coassembly process, as any structure disruption would lead to unwanted interactions between the components and unspecific aggregation of the system. Thus, a temperature at which the supramolecular interactions of the **E7** fibers are disrupted but in which the protein remains stable had to be chosen. The melting temperatures (*T*_m_) of the **C8-E7** and **C8-2E7** proteins were measured to be 67.6 °C (Figure S5B) and 70.1 °C (Figure S5C), respectively. Therefore, an annealing temperature of 60 °C was chosen for the coassembly. At this temperature, both proteins remained mainly folded and recovered fully the structure when cooled back to room temperature (RT) (Figure S5B,C). Meanwhile, the fibers were preserved, but the intermolecular interactions were weakened enough to permit the insertion of the peptide attached to the engineered CTPR8 protein.

### Proof of Concept and Optimization of the 1D Coassembled Fibers

The formation of 1D coassembled protein–peptide fibers is triggered by the inclusion of the peptidic sequence of the **C8-E7** protein into the **E7** fibers (Figure 4A). In this case, the coassembly is confined to one dimension by having only one **E7** peptide attached to the CTPR8 protein, limiting the interaction of the protein with one fiber.^72,88,89^ First, the protocol for the coassembly was optimized by considering annealing and sonication in different conditions (Figure S7). The final protocol consists of mixing **E7** (after 10 min of sonication) and **C8-E7** at a final concentration within the mixture of 1 mM and 71 μM, respectively, followed by 1 h annealing at 60 °C of the mixture. Using this protocol, TEM images (Figure 4C) showed a preponderance of fibers with a width of approximately 10–15 nm at 1:14 ratio (Figures 4D, S8A), corresponding to fibers decorated with proteins on only one side. In contrast, when the protein concentration in the coassembly was increased, using a 1:7 ratio, the majority of the fibers observed were around 20 nm in width (Figures 4D, S8A), in agreement with the theoretical dimensions expected (Figure 4B) for fibers with CTPR proteins on both sides. Note that in both coassemblies, three different population of fibers (**E7**, one-sided, and two-sided) are observed. However, the predominance of each population depends on the amount of protein used (Figure S8B). Furthermore, the formation of one-sided and two-sided coassembled fibers suggests that CTPR proteins also play a fundamental role in the organization of the proteins in the assembly. Their proximity, driven by the coassembly with the fibers, may force the proteins to self-assemble through side-to-side interactions in a similar way as they do in the CTPR-only thin films.^75^ These results validate the controlled coassembly between proteins and fibers, paving the way for the development of more complex coassembled systems.^75^

### Development of 2D Paracrystals through the Coassembly between Peptides and Protein–Peptide Complexes

**Figure 5:**
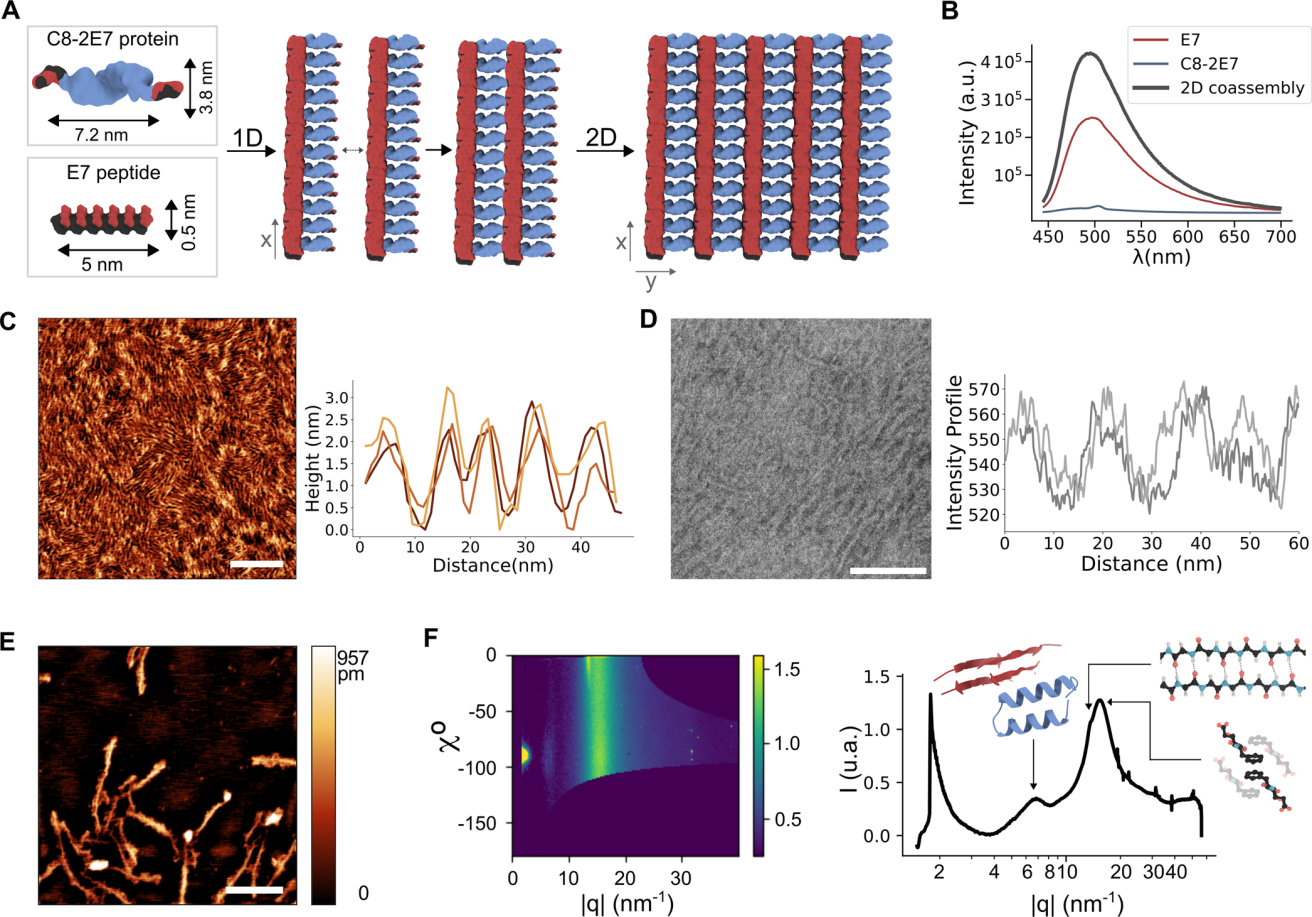
Design and characterization of the 2D coassembled paracrystals. A. Dimensions of the individual components and theoretical design of the 2D coassembly. B. ThT fluorescence assay in solution. C. AFM, and D. TEM characterization of the 2D system (top) with the corresponding measured profiles (bottom). E. AFM image of the disassembled system. F. Scattering patterns remapped as χ*c* vs |*q*| and the 1D azimuthally integrated intensity profile (right). All scale bars are 100 nm.

Pursuing the validation of the coassembly with the 1D coassembled system, the 2D system was subsequently explored using the **C8-2E7** engineered protein. The CTPR8 protein with two **E7** peptides fused can interact with two fibers simultaneously, acting as a cross-linker between fibers, which in combination with the self-assembly properties of the CTPR proteins was hypothesized to expand the system in the *x* and *y* dimensions. The stoichiometry of the 2D coassembled system matched that of the one-sided coassembled fibers (*i.e*., 1:14), as the 2D system is essentially the one-sided fibers repeated along the *y*-axis (Figure 5A).

As the low concentrations employed to favor the formation of the 2D assembly do not permit the use of FT-IR, and in CD the strong CTPR8 signal masks any contribution from the peptide assembly, alternative ways of monitoring the coassembly in solution between **C8-2E7** and the **E7** fibers were sought. Thioflavin T (ThT) is a fluorescence dye whose intensity is employed to monitor amyloid fibrilization and has also been extensively used for short peptide self-assembly.^91,92^ ThT was incubated with both peptide fibers and protein elements individually as controls and with the coassembled system. The results showed that the **E7** fibers exhibit the expected high fluorescence intensity for a self-assembled β-sheets-based supramolecular system. Second, the **C8-2E7** proteins did not show significant fluorescence, as expected due to the absence of aggregation and amyloid-like structure. These results corroborate the lack of any aggregated structure formation of the peptides within **C8-2E7** at this concentration, as observed by DLS (Figure S4E). Interestingly, the protein–peptide coassembly displayed an enhancement in intensity in comparison with the **E7** fibers (Figure 5B). This result suggests an increase in the peptide assembly through the aromatic residues, and a consequent increase in the amount of β-sheet order, consistent with the integration of the peptides of the **C8-2E7** into the **E7** fibers and confirming the formation of the coassembly in solution. Furthermore, the coassembly of both components does not alter the secondary structure of the protein, as confirmed by CD (Figure S9A). While the high α-helix content of the protein masks the ß-sheet signal of the fibers, the results confirm that the structure of the protein is preserved in the coassembly.

The structures formed were studied through AFM and TEM. Both techniques revealed that the morphology of the system shows an intended directionality that comes from the appropriate alignment of the fibers linked through the **C8-2E7** protein, revealing a paracrystalline-like structure (Figure 5C,D). The intensity profile from the TEM and the height profile from the AFM images display patterns with dimensions that lie within the range of the individual components of the coassembly (Figure 5A,C,D). Therefore, these experimental results demonstrate the formation of the proposed paracrystal lattice structure based on the extended cross-linking of the **E7** fibers with the **C8-2E7** protein in a 2D coassembly.

Different conditions (*i.e.*, concentrations, deposition procedures, and peptide/protein ratios) were tested to assess their impact on the system’s coassembly. First, employing an excess of fiber with a protein:peptide ratio of 1:20, did not trigger the formation of a uniform coassembled material. Instead, the fibers were randomly dispersed throughout the sample with no periodicity or apparent directionality (Figure S9B). Second, when the material was spin-coated at a low concentration, the system disassembled, revealing well-dispersed fibers with a “villi-like” morphology surrounding the fiber’s surface. This “villi-like” morphology might correspond to **C8-2E7** proteins attached to the fibers through only one of the two peptides, observing protein-decorated fibers (Figure 5E) rather than paracrystal coassemblies (Figure 5C). This observation supports that the proteins are correctly coassembled with the fibers. Overall, these two negative controls further validated the formation of the 2D paracrystal coassemblies under the designed conditions with the optimized protocol and ratio.

The influence on the morphology of the 2D system was studied by varying the deposition methods and concentration. First, the use of calcium ions in the drop-cast and spin-coated deposition of the material increased the film roughness by tens of nanometers (Figure S10A–C). The roughness variates from around 3.5 nm without the addition of calcium ions, to approximately 13.8 nm when deposited with calcium ions in the spin-coated systems (Figure S10B,C). This increased roughness might result from the positive ions acting as cross-linkers between the negatively charged fibers and proteins, inducing slight aggregation within the system. Interestingly, the calcium-free spin-coated films presented a morphology that resembled a crumpled paper, which might result from the twist and chirality of the fibers linked by a helical CTPR protein that does not lie flat either (Figure S10C). Lastly, spin-coating the material raised the critical deposition concentration by an order of magnitude, probably due to the loss of material during the spin-coating process and a disruptive effect in the assembly of the forces involved in this deposition method (Figure S10D). The disassembled systems displayed the fibers and proteins randomly dispersed, similar to the features observed in the individual controls of both components (Figure S10E). These results showed that the morphology and assembly of the films can be controlled by varying the deposition method and concentration of the system, in which the choice of methodology is dependent on the specific features desired.

The anisotropy of the material previously observed by TEM and AFM was further confirmed in deposited films by analyzing the atomic and molecular arrangements of the individual components within the system by Grazing-Incidence Wide-Angle X-ray scattering (GIWAXS), which is a technique commonly applied to study the crystalline phases and orientation of polymeric thin films.^93−97^ At a diluted concentration below the assembly threshold, the intensity profile did not show a detectable scattering pattern, suggesting that the system is disassembled and lacks any topological order (Figure S11). However, at a concentration above the assembly threshold, three peaks appeared in the profile. These signals appeared at *q* values of 15.35, 13.69, and 6.89 nm^–1^ (Figure 5F), reciprocal space values that can be easily transformed into real space distances (*D*) by applying the equation: . The peak at a *q* = 6.89 nm^–1^ corresponds to a real-space distance (*D*) of *D* = 9.1 Å. This distance is consistent with the periodic distance between the helices of the CTPR proteins but also with the intersheet separation of the ß-sheet peptides, as these distances around 10 Å are usually attributed in biological systems to the periodic separation between secondary structures.^98^ The peak at a *q* = 13.69 nm^–1^ corresponds to an atomic distance of 4.6 Å, which coincides with the hydrogen bonding distance.^98^ Therefore, this signal is compatible with the periodic repetition of the hydrogen bond interactions between the backbones of the α-helices of the CTPR proteins^75^ and the backbones of the ß-sheet peptides within the fibers. The last peak at *q* = 15.35 nm^–1^ corresponds to a *D* of 4 Å, which can correspond to the π-stacking of the phenylalanine side chains forming extended stacks at the core of the supramolecular fibers.^99^

Orientation information on the scattering features could be extracted from the 2D GIWAXS patterns by determining the azimuthal χ angle for a given |*q*|. In this case, features located at *χ* = 0° will have an in-plane orientation concerning the sample plane, while those present at *χ* = −90° originated from out-of-plane preferentially oriented crystallographic planes. Therefore, the scattering observed at |*q*| = 13.69 nm^–1^ and located at an angle close to 0° indicated that the hydrogen bonds planes were highly ordered, in this case, perpendicular to the substrate^99^ with an in-plane orientation (Figure 5F, left). Furthermore, the intensity of the signal corresponding to the interhelix and intersheet distances (*q* = 6.89 nm^–1^) consistently appeared at *χ* = −90°, suggesting that the drop-cased films arranged as 2D layers were preferentially stacked periodically, one on top of the other with an out-of-plane direction. In contrast, the other scattering band appeared across all χ angles, corresponding to the orientation of the π-stacking interactions through the three dimensions, likely due to the z-stacking of the 2D paracrystalline lattice layers. Thus, the scattering signals demonstrate the high paracrystalline topological order in the *x*/*y* planes, triggered by the coassembly between the proteins and peptides and the tendency of the individual components to self-assemble, as well as the stacking of the 2D layers in the *z*-plane.

The application of these materials as bioconductive films required optimal control over the thickness of their deposition. To first characterize and optimize the thickness of the material in the *z*-dimension, the effect of concentration and different deposition methods were studied. These measurements were performed by using native SiO_2_ substrates since the optical properties of this substrate allow to determine visually the homogeneity and thickness of the films quantitively, as recently studied by Almonte et al.^100^ Moreover, the thickness was also quantified by making an incision on the material and studying the height by AFM (Figure S12). The colors of the deposited films showed that spin-coating the material allows for the obtention of more homogeneous films than when drop-casted. Similarly, lowering the concentration in drop-casted films also enhanced the homogeneity of the films (Figure S12). Furthermore, the thickness of these deposited films could vary from 100 nm to even 10 nm in height. The highly concentrated drop-casted samples showed dimensions of around 100 nm in height, as observed in the AFM profile (Figure S12A). Meanwhile, the highly concentrated spin-coated (Figure S12B) and the low concentrated drop-casted (Figure S12C) samples could reach thicknesses of around 10 nm height. Considering the dimensions of the individual systems, a single layer of the 2D paracrystal would range from 5 to 10 nm. However, this crumpled morphology could increase these theoretical dimensions, and these 10 nm films might consist of one or, at most, two monolayers of the coassembled material. Thus, these results demonstrate that such highly ordered materials enable precise control over film thickness, allowing the formation of materials around 100 nm, as well as thin films as small as 10 nm.

### Ionic Conductivity in 2D Coassemblies

CTPR-based materials have previously been shown to exhibit incipient protonic conductivity,^47^ likely enhanced by the presence of charged amino acids. Moreover, the **E7** peptide displays 7 glutamic acids, mainly on the surface of the supramolecular fibers, which may contribute to proton transport. This feature endows both the **E7** peptide fiber assemblies and the **C8-2E7** protein films with significant potential for proton conduction. Consequently, the synergy between these systems positions the 2D coassembled supramolecular structure as a promising candidate for efficient ionic conductivity within a highly ordered framework.

**Figure 6:**
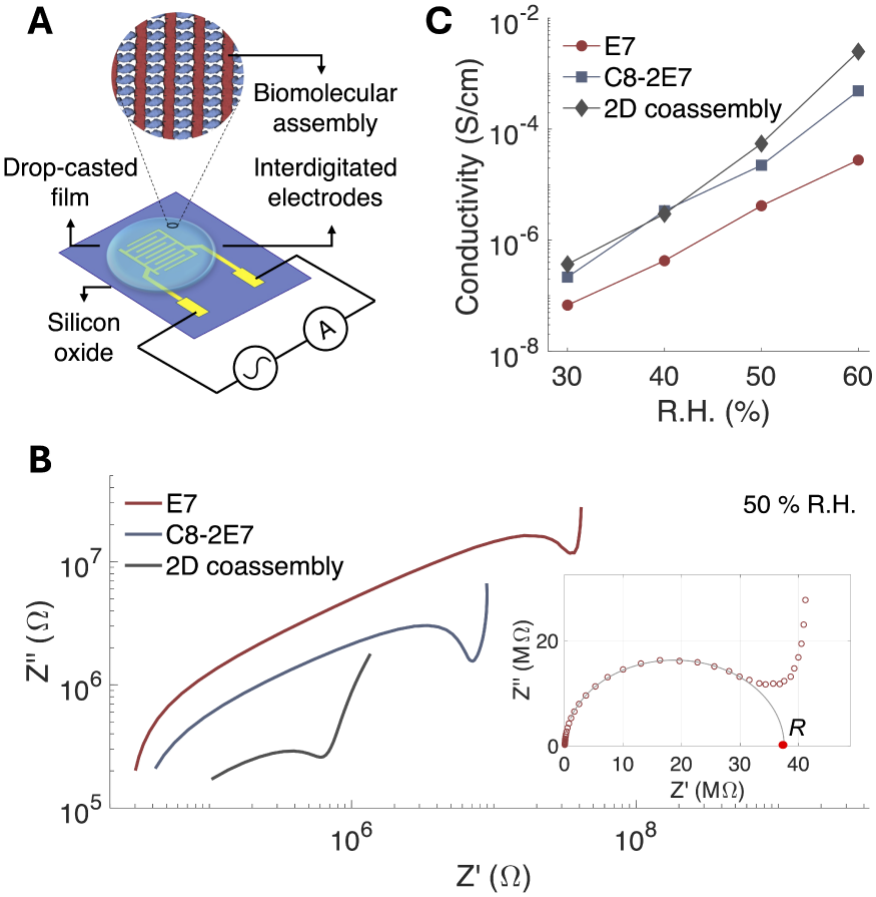
Electrical characterization of the E7 fibers, C8–2E7 films, and the 2D coassembled films. A. Schematic representation of the experimental device, illustrating the 2D coassembled film deposited on an oxidized silicon substrate with gold interdigitated electrodes for EIS characterization. B. EIS Nyquist plots for **E7** fibers, **C8-2E7**, and the 2D coassembly measured at 50% relative humidity. The data is presented on a logarithmic scale for comparison. The inset shows the EIS data for **E7** fiber assembly on a linear scale. Impedance data (red empty circles) is fitted to an elliptical function (gray line) and the bulk ionic resistance of the material (R) is determined from the ellipse’s intercept with the *Z*′-axis (marked with a solid red dot). C. Conductivity as a function of relative humidity, ranging from 30 to 60%, for **E7** fibers, **C8–2E7** protein films, and the 2D coassembly.

For the electrical characterization of the systems, films of **E7** fibers, **C8-2E7**, and their 2D coassembly were prepared on top of prepatterned interdigitated electrodes using the drop-casting method (see Methods and Figure 6A). The conduction properties of the films were investigated using electrical impedance spectroscopy (EIS), a well-established technique for studying the electrical response of biomaterials.^35^ The resulting impedance data are graphically represented in Nyquist diagrams (Figure 6B) for the three different materials (**E7**, **C8-2E7**, and 2D coassembly) at a fixed relative humidity RH = 50%. Typical Nyquist diagrams for ionic conductors with blocking electrodes exhibit a depressed semicircular region, which is associated with the bulk response of the material, followed by a tilted spike that arises from the diffusion effects at the electrode interface. From the Nyquist diagrams, the bulk ionic resistance of the samples can be extracted by fitting the semicircular region to an elliptical function and identifying its intersection with the real axis (*Z*’), as shown in the inset of Figure 6B. The comparison of the Nyquist plots in Figure 6B for **E7** fibers, **C8-2E7** protein, and 2D coassembly at 50% RH revealed that the 2D coassembly exhibited a significantly lower resistance, approximately 1 MΩ compared to **E7** fibers and **C8-2E7** protein, which showed resistances of around 100 MΩ and 10 MΩ, respectively (Table S3). Likely due to differences in the assembly characteristics of each biomolecular compound, their films showed different thicknesses. A thickness estimation based on the analysis of the differential reflectance of the films^100^ (see Methods and Figure S13) indicated that the 2D coassembly samples exhibited an average thickness of 280 ± 60 nm while **E7** and **C8-2E7** films were thinner, with thicknesses of approximately 90 ± 20 nm and 100 ± 10 nm, respectively (Table S2). Therefore, a fair comparison of the electrical properties of the different samples required the calculation of their conductivities. The conductivity of the samples was calculated using the following expression:^101^
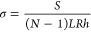
where σ represents conductivity, *S* is the distance between the electrodes, *L* is the length of the electrode, *N* is the number of electrodes, *h* is the sample’s thickness, and *R* is the film’s ionic resistance obtained from the analysis of the Nyquist diagrams.

At fixed relative humidity (50%), **C8-2E7** films exhibit conductivity values (σ ∼ 1.8 × 10^–5^ S/cm), which are comparable to those of their CTPR8 counterparts (see Figures S14, S16 and Table S4). Notably, the 2D coassembly exhibits distinct structural properties compared to the CTPR self-assembled films while retaining the efficient ionic conductivity of CTPR assemblies, with conductivity values σ ∼ 5.5 × 10^–5^ S/cm. More remarkably, the ionic conductivity of the 2D coassemblies is an order of magnitude higher than that of peptide-only **E7** fiber assemblies (σ ∼ 4.2 × 10^–6^ S/cm). These findings indicate that the structured coassembled material is a significantly more efficient ionic conductor than the nonordered peptide fiber assemblies. This trend is consistent across different relatively humid conditions ranging from 30 to 80% (Figure 6C and Table S4). All three materials show exponential increases in conductivity with rising humidity, which is a common trend in biomaterials, particularly in protein-based materials, consistent with ionic conductivity dominated by proton conduction.^102^

In summary, these results highlight that the integration of **E7** fibers with the **C8-2E7** protein into a 2D paracrystal results in a coassembled structure that incorporates the ionic conductivity characteristics of the CTPR systems to the peptide assemblies, reaching maximum values in high-humidity environments.

## Conclusions

In conclusion, this work provides a framework for biomolecular design of peptide- and protein-assembled systems. First, strategies to fine-tune the length and size of peptide sequences to develop self-assembled supramolecular fibers are presented, identifying the **E7** peptide as the optimal sequence for the stable coassembly with proteins. Second, the fusion of one (**C8-E7**) or two previously optimized amphiphilic peptides (**C8-2E7**) to a superhelical repeat protein demonstrated that these peptides drive the coassembly between the modified proteins and the peptide self-assembled fibers. This approach paves the way to constructing intricate supramolecular structures through spontaneous assembly of distinct building blocks, yielding materials with tailored and precise dimensionalities, *i.e*., 1D coassembled fibers and 2D coassembled paracrystals. The 2D paracrystals exhibited nanoscale anisotropic order characteristic of a peptide–protein coassembled system, which is absent in the disassembled systems. More remarkably, these 2D paracrystals demonstrated exceptional potential for controlled thin-film fabrication, achieving thicknesses as low as 10 nm when deposited on a substrate. Furthermore, these 2D coassemblies exhibited effective ionic conductivity, a property that is absent in peptide-based self-assembled fiber films. Notably, this functionality was achieved without the need for nonbiological moieties which are typically required for efficient conduction, underscoring the inherent capabilities of the biomolecular design. This work showcases the integration of structural precision and functionality, charting new directions for the development of nanostructured materials and bioelectronic platforms. This research could broaden the potential for advancements in various research directions, such as integrating nanoparticles for signaling or functionalizing substrates with nanoscopic resolution. Additionally, proteins that modulate cellular responses could be incorporated into the coassemblies, transforming these materials into bioactive platforms for controlling cell behavior in which the ordered morphology of the material could lead to an enhanced interaction with the cells. These innovations could pave the way for a wide range of applications, from tissue engineering to next-generation bioelectronics, opening novel opportunities for future research and technological development.

## Methods

### Molecular Dynamics Simulations

The coarse-grained simulations were performed using the Martini 2.2 CG force field^103^ and the GROMACS software (version 2022).^104^ The structures of the peptides were built using Avogadro 1.2^105^ and transformed to MARTINI resolution using martinize.py script,^106^ using extended structure (ß-sheet) (E) for secondary structure. For all the simulations the number of peptides randomly inserted in a 15 nm^3^ box was 80 peptides, resulting in a final concentration of 39.36 mM. The systems were solvated with explicit Martini CG water, neutralizing the system’s charge with Na^+^ and Cl^–^ ions. The minimum distance between molecules was 3 Å and 2 Å between water molecules. A minimization step was performed using the steepest decent minimization algorithm setting a maximum force threshold of 2 × 10^3^ kJ/mol/nm. For the screening of the dipeptides, the simulations (Figure 1C) and the screening of the linkers (Figure 3B,C) were run for 2 μs, and for the study of the negative charges (Figure 1E,F) they were run for 5 μs, using a 25 fs time step and applying the 4× time factor correction. The simulations were performed at the *NPT* ensemble. The temperature was set at 303 K using the v-rescale thermostat with a τ_T_ of 1 ps. The pressure was set at 1 bar using the Berendsen algorithm with a τ_p_ of 1 ps through isotropic coupling.

The visualization of all the structures and rendering of the images was performed using Chimera X 1.6.1.^107^

The aggregation propensity (AP) analysis was calculated using the methodology described by Frederix *et al.*,^58^ using the solvent-accessible surface area (SASA) calculated during the last 0.5 μs of the simulations. The water contacts are the coordination number (*g*(*r*)) at 0.7 nm of water beads with each backbone bead during the last μs (Figures 1C and 3B,C). The average in Figure 1E excludes the N-terminal and the C-terminal beads. The average analysis in Figure 3B is done on the linker sequence for each peptide without considering the **E7** sequence.

### Fiber Preparation

The concentration of the peptides was 4 mM for all the peptides except for **E6** which was prepared at 2 mM given that at 4 mM the gel is too viscous. The samples were prepared in water at pH 5 sonicated for 10 min and followed by annealing at 80° for 30 min in a water bath, letting the system reach room temperature overnight.

### Molecular Biology

A Polymerase Chain Reaction (PCR) was performed using the CTPR8 DNA template and the primer containing the DNA of the **E7** sequence to obtain the corresponding DNA of the **C8-E7** protein. The primer sequence was ordered to Sigma-Aldrich. The Phusion High-Proof DNA Polymerase was used to insert the **E7** sequence to the CTPR8 template performing the PCR at 57.3, 60.1, 62.4, and 64 °C. This insert was ligated to a pProEx prokaryotic expression vector with resistance to ampicillin and the IPTG (isopropyl ß-d-thiogalactoside) inducible promoter. The ligation was performed using a 1:4 ratio of plasmid to insert. D10ß *Escherichia coli* cells were transformed with the resulting plasmid. The DNA of the **C8-2E7** was obtained by digesting the plasmid of the **C8-E7** with NcoI and *Bam*HI restriction enzymes, which cut the sequence between the His-Tag and the CTPR sequence and ligating the inset containing the **E7** sequence to this plasmid. The protein sequences can be found in Table 1.

**Table 1:** Amino Acid Sequence of the Designed Proteins (**C8-E7** and **C8-2E7**)

Protein	Sequence
**C8-E7**	GSAEAWYNLGNAYYKQGDYDEAIEYYQKALELDPRSAEAWYNLGNAYYKQGDYDEAIEYYQKALELDPRSAEAWYNLGNAYYKQGDYDEAIEYYQKALELDPRSAEAWYNLGNAYYKQGDYDEAIEYYQKALELDPRSAEAWYNLGNAYYKQGDYDEAIEYYQKALELDPRSAEAWYNLGNAYYKQGDYDEAIEYYQKALELDPRSAEAWYNLGNAYYKQGDYDEAIEYYQKALELDPRSAEAWYNLGNAYYKQGDYDEAIEYYQKALELDPRSAEAKQNLGNAKQKQGEFGGSSEEEEFEFEFEFEFEFE-
**C8-2E7**	EFEFEFEFEFEFEEEESSGGGSAEAWYNLGNAYYKQGDYDEAIEYYQKALELDPRSAEAWYNLGNAYYKQGDYDEAIEYYQKALELDPRSAEAWYNLGNAYYKQGDYDEAIEYYQKALELDPRSAEAWYNLGNAYYKQGDYDEAIEYYQKALELDPRSAEAWYNLGNAYYKQGDYDEAIEYYQKALELDPRSAEAWYNLGNAYYKQGDYDEAIEYYQKALELDPRSAEAWYNLGNAYYKQGDYDEAIEYYQKALELDPRSAEAWYNLGNAYYKQGDYDEAIEYYQKALELDPRSAEAKQNLGNAKQKQGEFGGSSEEEEFEFEFEFEFEFE-

### Protein Expression and Purification

The C41 *E. coli* cells were transformed overnight at 37 °C with the plasmid containing thesequences for the expression of **C8-E7** or **C8-2E7** and cultured in LB medium with ampicillin. Protein expression was induced when the OD_600_ was 0.6–0.7 with 0.6 mM IPTG and the culture was grown overnight at 20 °C. Cells were resuspended in urea lysis buffer (500 mM urea, 500 mM NaCl, 50 mM Tris, 5 mM imidazole, pH 8) with lysozyme (1 mg/mL), DNase (0.2 μL/mL), and 1/5 tablet of EDTA-free protease inhibitor per liter of cell culture. For the **C8-E7** protein, the lysate was sonicated at 4 °C in two cycles of 10 min with 1 s intervals. Centrifugation at 10,000 rpm for 45 min was performed to separate the soluble and insoluble fractions. The protein was purified from the supernatant by using a nickel HisTrap column, washing with 10 mM imidazole, and eluting with 300 mM imidazole. The His-tag was removed with TEV protease in 1 mM DTT, 0.5 mM EDTA, and 10% glycerol. Given the increase in the hydrophobicity of the **C8–2E7** protein, the protein was purified from the insoluble fraction after centrifugation at 10000 rpm, by redissolving the pellet in 6 M urea lysis buffer to break these inclusion bodies, provoking protein unfolding. The lysate was sonicated and centrifugated again in the same conditions. Afterward, the supernatant was incubated with nickel-charged affinity resin (Ni-NTA) for 48 h at 4 °C. Through gravity flow purification the urea concentration was linearly decreased by 1 M at each round to recover the protein’s native conformation. Once the protein attached to the column was in lysis buffer w/o urea, it was also washed with 20 mM imidazole and eluted with 300 mM imidazole. An ion exchange protein purification was performed after the His-tag cleavage by using a HiTrapQ column, applying a salt gradient with a start buffer (20 mM Tris) and an elution buffer (20 mM Tris, 1 M NaCl) to obtain a pure protein (Figure S4B).

### Protein–Peptide Coassemblies

The **E7** fibers were annealed as reported in the “Fiber Preparation” section at 4 mM. These fibers were then sonicated for 10 min. The **C8-E7** or **C8-2E7** proteins in Tris 10 mM and NaCl 10 mM buffer at pH 7.4 for the characterization in Figures 4 and 5, or Tris 0.5 mM and NaCl 0.5 mM buffer at pH 7.4 for the electronic characterization in Figure 6, were added to the sonicated batch of **E7**. The final concentration of the assemblies was 1 mM peptide and 71 μM protein for a 1:14 ratio. Note that the concentration of the protein changes depending on the ratio and the concentration of the peptide remains constant. Then the mixtures were annealed for 1 h at 60 °C.

### Circular Dichroism

A Jasco J-815 spectrophotometer was used to analyze the secondary structure of the different systems. A quartz cuvette with an optical path of 1 cm was used for the measurements The data is represented as molar ellipticity (Δε), being *c* the concentration (in M), and *l* the optical path length (in cm);
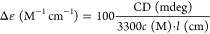


The fiber samples were prepared at 25 μM in water at pH 5. The CD spectra were recorded from 300 to 190 nm at RT with a 0.2 nm data pitch using a continuous scanning speed of 50 nm/min and 5 accumulations. The Digital Integration Time (DIT) was set at 1 s, and the bandwidth at 1 nm.

### Fourier-Transform Infrared (FT-IR)

An Invenio-X Bruker FT-IR was used for the measurements, placing the samples between two CaF_2_ windows of 32 nm, separated by a Teflon spacer of 50 μm. The measurements were recorded by accumulating 25 scans with a 1 cm^–1^ resolution. The data was analyzed by subtracting the relative absorptions of D_2_O. The data was normalized by the value of the hydrogen bonding to compare between the samples. The concentration of the samples was 10 mM in D_2_O at pH 5.

### Fluorescence Spectroscopy

The fluorescence data was obtained using an FS5 spectrofluorometer (Edinburgh Instruments). The data was recorded from 445 to 700 nm for 5 repeats, with a fixed wavelength offset at 430 nm. The **E7** peptide was measured at 100 μM, the **C8-2E7** protein at 7.1 μM, and the 2D paracrystal at the corresponding concentrations of 100 μM peptide and 7.1 μM protein. The concentration of ThT was 0.5 μM for all the samples.

### Transmission Electron Microscopy (TEM)

TEM images were acquired with an EOL JEM-1400PLUS (40–120 kV, HC pole piece) LaB6-TEM equipped with a GATAN US1000 CCD camera (2k × 2k). To visualize the samples negative staining was used. For the fibers in Figures 2B and S2, the samples were deposited at 10 μM on top of 400 Mesh Copper (100) carbon films from EM Resolutions, stained with uranyl acetate 1.5%, and followed by 2 washes with water. For the coassemblies in Figures 4C, 5D, S7, S8A and S9, the samples were deposited on top of Ultrathin C films on Lacy Carbon from Ted Pella at 10 μM of the peptide and the corresponding concentration of the proteins according to the different ratios, then the samples were stained with uranyl acetate 0.5% followed by 1 wash with water. The images were processed and analyzed using the Fiji^108^ Software.

### Atomic Force Microscopy (AFM)

The images were acquired using a Multimode 8 HR-U Veeco Bruker AFM with the NanoscopeV controller. The measurements were performed in dry tapping mode using the OTESPA-R3 aluminum-coated cantilever (*f*_o_ = 300 kHz). For the characterization of the fibers and the 2D paracrystal in Figures 2A and 5C the samples were deposited at 1 mM on top of a mica substrate, then washed with 150 mM NaCl_2_ and 50 mM CaCl_2_ and dried overnight. For the characterization of the disassembled 2D paracrystal in Figure 5E, the sample was deposited at 10 μM peptide 0.71 μM protein and spin-coated. The data visualization and image analysis were performed with the Gwyddion^109^ Software.

### Grazing Incidence Wide Angle X-ray Scattering (GIWAXS)

This synchrotron-based technique is a powerful tool used to investigate the structural organization of thin films and surfaces at the molecular and nanoscale levels. Surface sensitivity is achieved by directing the X-ray beam at a shallow angle to the sample surface, near the surface critical angle. The recorded 2D scattering patterns correlate with the surface orientation, structure, and composition, enabling the study of the atomic and molecular distances and orientation within crystal lattices. In this case, GIWAXS data was acquired at the ALBA synchrotron in the NCD-SWEET beamline (Cerdanyola del Vallès, Spain). A 12.4 keV monochromatic X-ray beam (*l* = 0.9998 Å) of 150 × 150 μm^2^ [*H* × *V*] was prepared using a Si(111) channel-cut monochromator and collimated with an array of Be lenses. The scattered light was recorded with a Rayonix LX255-HS area detector placed at 180.62 mm from the sample position. Cr_2_O_3_ was used as a calibrant to calculate sample-to-detector distance and detector tilts. To ensure surface sensitivity, the grazing incidence frames were recorded at incident angles (α_i_) between 0.1° and 0.16° for 1, 5, 10, and 30 s of integration time. The momentum transfer (*q*) refers to the change between the incident and the scattered ray’s momentum, providing information on the periodic orientation of particles within thin films.^97,110^ The reciprocal maps were remapped to the azimuthal angle (χ) vs |*q*| to simplify the orientation analysis and remapped to represent 1D profiles using PyFAI.^111^ The data was imported, processed, and the plots were represented using Python. Note that for the interpretation of Figure 5F, χ is the azimuthal angle concerning the sample surface: χ = −90° means the normal surface direction, while χ = 0° means an in-plane orientation, *i.e.*, crystallographic planes perpendicular to the surface plane. The plotted data corresponds to the sum of the four images at each integration time, at the incident angle α_i_ = 0.12°. For each image, the background was subtracted and normalized by the corresponding incoming intensity at the time of the acquisition. The relation between the momentum transfer (*q*) and atomic distance (*D*) is .

### Sample Preparation for Electrical Characterization

Ti/Au (5 nm/40 nm) interdigitated electrodes (IDEs) were fabricated using optical lithography on Si/SiO_2_ substrates with a 295 nm oxide layer. The electrodes had a track width (*W*) of 25 μm and an interelectrode spacing (*S*) of 25 μm. Each IDE array consisted of 21 electrodes (*N*), with an electrode length (*L*) of 1020 μm. The substrates were cleaned by sonication in acetone and isopropanol and subsequently dried using nitrogen flow. To improve surface hydrophilicity, a 30 s argon plasma cleaning was applied to the substrates. Immediately after, 0.5 μL of a solution containing **E7** fibers (1 mM), CTPR8 protein (71 μM), **C8-2E7** (71 μM), or the coassembly (**E7** fibers 1 mM and **C8-2E7** 71 μM), was drop-casted at the center of the IDE using a micropipette. After deposition, the samples were left to dry under ambient conditions (25 °C and 51% relative humidity) for 24 h.

### Microreflectance Spectroscopy Thickness Determination

Microreflectance spectroscopy was employed to estimate the thickness of the 2D coassembly, the **C8-2E7**, the CTPR8 protein, and **E7** fibers films using a modified microscope following the method proposed in ref (100). White light from a halogen lamp was used to illuminate the sample, and the reflected light collected from a spot of ∼3 diameter was analyzed in a compact spectrometer. The reflectance spectrum was recorded from both the bare Si/SiO_2_ regions and the film-covered areas between the 25 μm electrode tracks. The optical contrast is as μμ, where *I*_films_ and *I*_subst_ represent the reflected intensities from the film and substrate, respectively (Figure S13). Film thickness was estimated by simulating optical contrast using a model based on Fresnel equations for light propagating in optical multilayer systems. The refractive index values for CTPR protein films were taken from ref (112) and for Si and SiO_2_ from ref (113). A constant SiO_2_ thickness of 295 nm was assumed. Thickness was estimated as the mean from four different measurements for each sample at different positions.

### Electrical Impedance Spectroscopy Characterization

Electrical Impedance Spectroscopy (EIS) measurements were performed as a function of relative humidity in a custom climatic chamber, following the same protocol for all samples. The process started at 30% RH and increased to 80% RH, with EIS measurements taken at 10% RH increments. Each sample was exposed to each humidity level for 15 min to equilibrate before the EIS measurements were performed.

EIS measurements were performed by applying a 10 mV AC excitation voltage to the sample with a frequency sweeping from 1 Hz to 10 kHz. The impedance response of the sample was recorded using a Stanford Research SR830 lock-in amplifier.

To calculate the bulk resistance of samples, impedance Nyquist plots were analyzed. For Nyquist plots showing a clear semicircular region, that region of data was fitted to an elliptical function. The intercept of that ellipse with the *X*-axis (*Z*′), is interpreted as the bulk ionic resistance of the sample, as graphically shown in the inset of Figure 6B. For measurements at the highest humidity levels, only the diffusion region of the impedance could be observed within the frequency range used. In these cases, the diffusion tail was fitted to a linear expression, and the resistance was determined from its intercept with the real impedance axis (Figure S15 and Table S3). This method underestimates the resistance of the samples so that the values obtained from this method can be considered a low-bound estimate of the actual resistance.

## Supporting Information Available

Characterization of the self-assembled fibers (widths calculation by TEM); propensity aggregation of the linkers (CG-MD simulations); protein characterization; thermal characterization of **E7**, **C8-E7**, and **C8-2E7**; average diameter of **E7** self-assembled fibers; characterization of the 2D coassembled system (ratio and protocol optimization, AFM characterization, GIWAXS of the disassembled system, and thickness characterization of the films by AFM); ionic conductivity in 2D assemblies (thickness of deposited films, Nyquist plots, resistance, and conductivity of the **E7** fibers, CTPR8, and **C8-2E7** protein films, and the 2D coassembled paracrystal) (PDF)

## Abbreviations

CG-MD: coarse-grained molecular dynamics
AA-MD: all-atom molecular dynamics
AP: aggregation propensity
SASA: solvent accessible surface area
TEM: transmission electron microscopy
AFM: atomic force microscopy
CD: circular dichroism
FT-IR: Fourier-transform infrared spectroscopy
CTPR: consensus tetratricopeptide repeat
RMSD: root-mean-square deviation
MALDI-TOF: matrix-assisted laser desorption/ionization time-of-flight
DLS: dynamic light scattering
*R*_h_: hydrodynamic radius
*T*_m_: melting temperature
GIWAXS: graze-incidence wide-angle x-ray scattering
EIS: electrical impedance spectroscopy
