# Supplementary material for: Tuning the Dimensionality of Protein–Peptide Coassemblies to Build 2D Conductive Nanomaterials
Source: ACS Nano. 2025 Apr 25;19(17):16500–16. doi: 10.1021/acsnano.4c18613 (PMC12060649; doi:10.1021/acsnano.4c18613)
Supplement: Supplementary file 1 — nn4c18613_si_001.pdf [file nn4c18613_si_001.pdf]

Supporting Information.

Tuning the dimensionality of protein-peptide coassemblies to build 2D conductive nanomaterials.

Laura Perez-Chirinos,[1] Lisa Almonte,[2,3] Juan David Cortés-Ossa,[2,3] Eduardo Solano,[4] M. Reyes Calvo,[2,3,6] Ivan R. Sasselli,[1,5] Aitziber L. Cortajarena.[1,6*]*

[1]Center for Cooperative Research in Biomaterials (CIC biomaGUNE), Basque Research and Technology Alliance (BRTA), Paseo de Miramón 194, 20014, Donostia-San Sebastián, Spain.

[2]Instituto Universitario de Materiales de Alicante (IUMA), Universidad de Alicante, Alicante 03690, Spain.

[3]BCMaterials, Basque Center for Materials, Applications and Nanostructures, UPV/EHU Science Park, Leioa, Vizcaya 48940, Spain

[4]NCD-SWEET Beamline, ALBA Synchrotron Light Source, 08290 Cerdanyola del Vallès, Barcelona 08290, Spain.

[5]Centro de Física de Materiales (CFM), CSIC-UPV/EHU, Paseo Manuel de Lardizabal 5, 20018, Donostia-San Sebastián, Spain.

[6]IKERBASQUE, Basque Foundation for Science, Plaza Euskadi 5, 48009 Bilbao, Spain.

Table of contents

Characterization of self-assembled fibers 4

Figure S1. Snapshots of the last frame of the CGMD simulations 4

Figure S2. Width of the self-assembled fibers 5

Propensity aggregation of the linkers 5

Figure S3. CG simulations of the E7 and L-E7 coassemblies at 1:3, 1:7, 1:14, and 1:30 ratios 5

Table S1. Number of E7 and L-E7 peptides inserted in the simulation box for each of the ratios in the coassembly CG-MD simulations 6

Protein characterization 6

Figure S4. Characterization of the CTPR8 and the C8-E7 and C8-2E7 engineered proteins 8

Thermal characterization of E7 fibers, C8-E7 and C8-2E7 9

Figure S5. Thermal denaturation and renaturation curves 9

Average diameter of the E7 fibers 10

Figure S6. Characterization of the E7 fiber 10

Characterization of the 2D coassembled material 10

Figure S7. Protocol optimization for the coassembly 12

Figure S8. TEM and AFM characterization of the 1D coassemblies 13

Figure S9. Validation of the protein stability within the assembly and optimal ratios 14

Figure S10. AFM characterization of the 2D co-assembly at varying substrates, dilutions and deposition methods 16

Figure S11. GIWAXS characterization of the disassembled 2D material at a 1:100 dilution 16

Figure S12. Thickness of the 2D co-assembled films 16

Ionic conductivity in 2D coassemblies 17

Figure S13. 17

Table S2. Thickness values for the E7 fibers, CTPR8, C8-2E7, and 2D paracrystal films 17

Figure S14. Nyquist plots of impedance spectra for three different materials 18

Table S3. Resistance values of E7 fibers, CTPR8, C8-2E7, and 2D paracrystal films 18

Figure S15. Nyquist plot of the impedance data for the CTPR8 protein 19

Table S4. Conductivity values of E7 fibers, CTPR8, C8-2E7, and 2D paracrystal films 19

Figure S16. Conductivity as a function of relative humidity 20

Characterization of self-assembled fibers

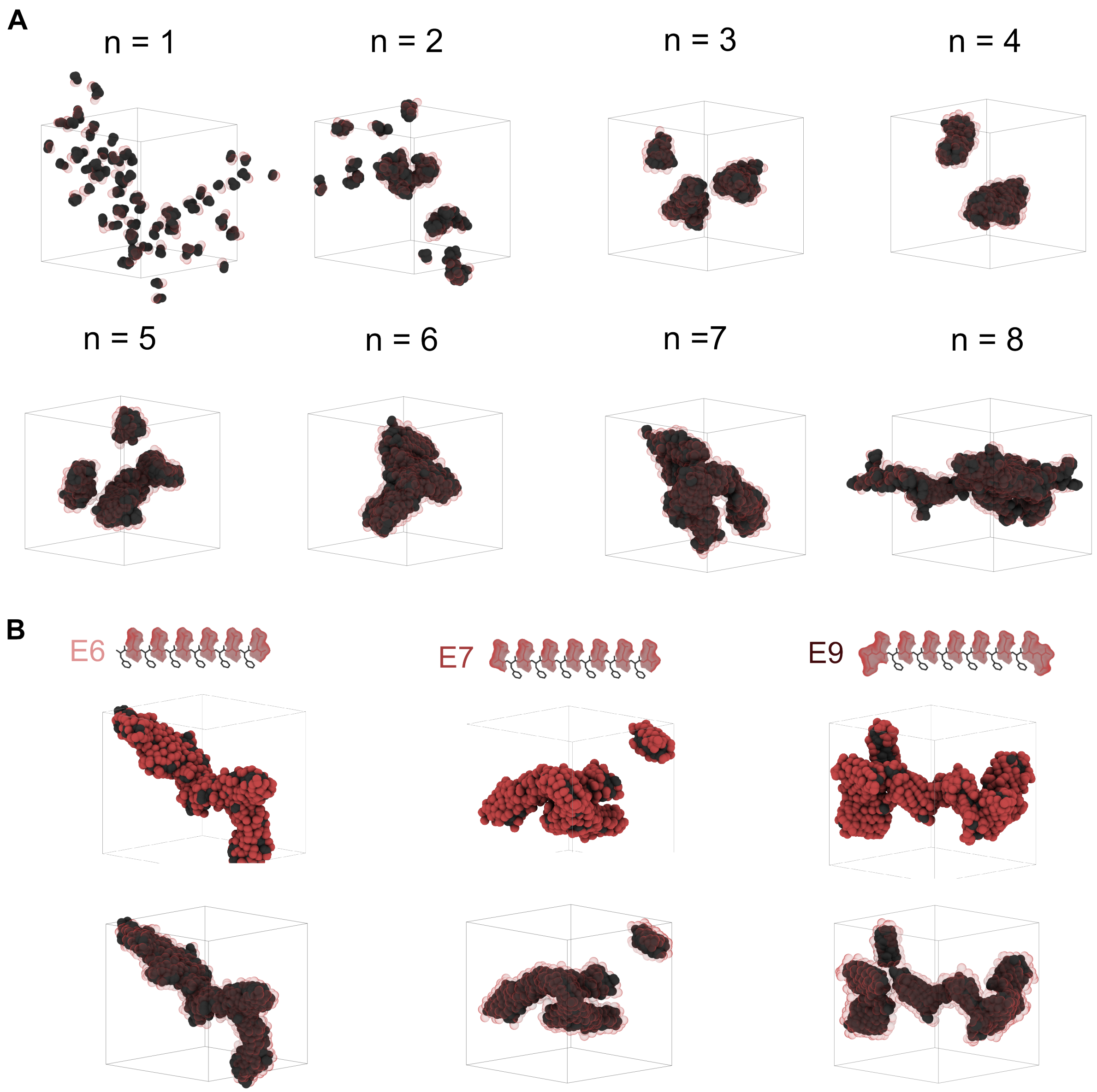

Figure S1. Snapshots of the last frame of the CGMD simulations. A. Snapshots of the simulations of the peptides from 1 to 8 repetitions of the $(FE)_n$ dipeptide with the glutamic beads in transparent. **B.** Snapshots of the simulations of the **E6**, **E7**, and **E9** peptides. Full colored beads (top). Glutamic beads in transparent (bottom). The phenylalanine residues are colored in black and the glutamic acid residues in red.

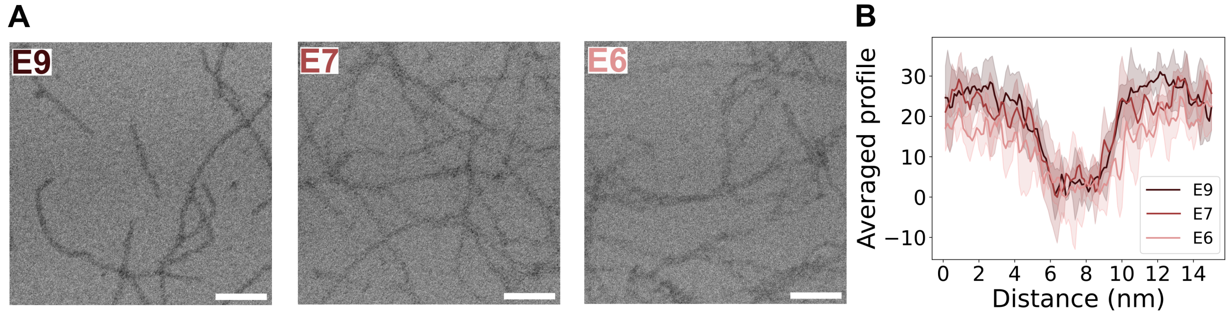

Figure S2. Width of the self-assembled fibers. A. TEM images of the **E9**, **E7**, and **E6** self-assembled fibers. Scale bars are 50 nm. **B.** Averaged profile from four different measurements of the TEM images.

Propensity aggregation of the linkers

CG MD simulations. The simulations of the Figure S3 were performed as described in the paper (see Materials and Methods). The simulations were run for 2 µs. The number of molecules inserted in a 20 × 20 × 20 nm simulation box is described in Table S1. In all the four cases the total final concentration is 39.4 mM.

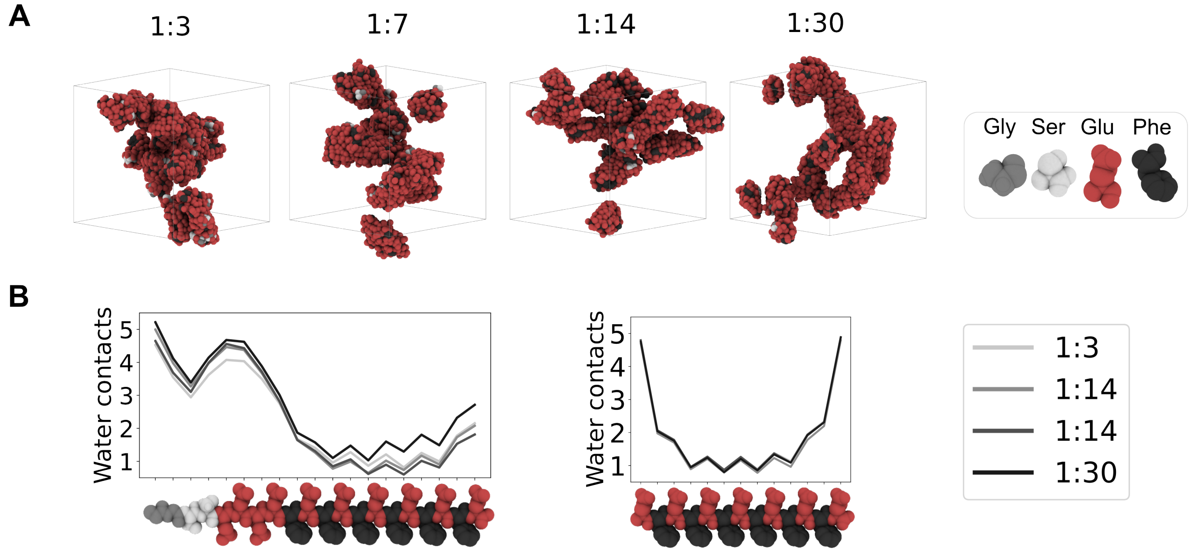

Figure S3. CG simulations of the E7 and L-E7 coassemblies at 1:3, 1:7, 1:14, and 1:30 ratios. A. Snapshot of the last frame of the simulations. **B.** Water contacts of the **L-E7** (left) and **E7** (right) for each ratio.

Table S1. Number of **E7** and **L-E7** peptides inserted in the simulation box for each of the ratios in the coassembly CG-MD simulations.

	**Ratio 1:3**	**Ratio 1:7**	**Ratio 1:14**	**Ratio 1:30**
**E7**	143	166	177	184
**L-E7**	47	24	13	6

Protein characterization

Atomistic MD simulations. The simulations of the Figure S4A were performed using the CHARMM26 force field[1,2] and the GROMACS software (version 2019).[3] The CTPR8 (PDB 2AVP)[4] crystal structure was used as the template for the simulations. The initial structure for the **C8-E7** and **C8-2E7** was built using the PDB of the CTPR and the **E7** peptide structure and linked using VMD.[5] One protein was inserted in a 17.08 nm^3 box. The system was solvated explicitly using the TIP3P water model. The ionic concentration of the systems was set to 150 mM using Na^+ and Cl^- ions to mimic physiological conditions. A first step of minimization was performed, setting a force threshold of 10^3 kJ/mol/nm using the steepest decent minimization algorithm. Using the NVT ensemble the systems were equilibrated for 5×10^4 steps with a 2 fs time step at 300 K using the v-rescale thermostat and a τ_T of 0.1 ps to keep the temperature constant. After the NVT equilibration, using the NPT ensemble the systems were equilibrated for 5×10^5 steps with a 2 fs time step using the Berendsen algorithm to keep the pressure constant at 1 bar with a τ_p of 2 ps, and the same conditions for the temperature than for the NVT equilibration. The simulations were performed for 100 ns with a 2 fs time step at the NPT ensemble, under isotropic conditions using the Parrinello-Rahman algorithm to keep the pressure constant with a τ_p of 2 ps. The temperature was set at 300 K with a τ_T of 0.1 ps using the v-rescale thermostat. The RMSD was calculated only taking into account the section corresponding to the CTPR protein.

Matrix Assisted Laser Desorption/Ionization time-of-flight (MALDI-TOF). The spectra of the corresponding proteins were obtained with a MALDI/TOF-TOF MS UltrafleXtreme III mass spectrometer (Bruker). The proteins were deposited on top of a MALDI plate, mixing 1 µL of

protein in 10 mM Tris and 10 mM NaCl buffer with 4 µL of matrix (10 mg/mL synaptic acid in a batch solution of ACN and 5% TFA).

Circular Dichroism (CD). The general measurements were performed as described in the Materials and Methods section. For the proteins the samples were prepared at 5 µM in 10 mM NaCl 10 mM Tris at pH 7.4. The CD spectra was recorded from 260 nm to 190 nm at RT with a 0.1 nm data pitch using a continuous scanning speed of 50 nm/min and 3 accumulations. The Digital Integration Time (DIT) was set at 1 second, and the bandwidth at 5 nm.

Dynamic Light Scattering (DLS). The hydrodynamic radius (R_h) of the proteins was obtained with a Malvern Zetasizer Ultra Red ZSU3305 instrument with a 633 nm laser. The concentration of the proteins was 5 µM in 10 mM Tris and 10 mM NaCl buffer at pH 7.4.

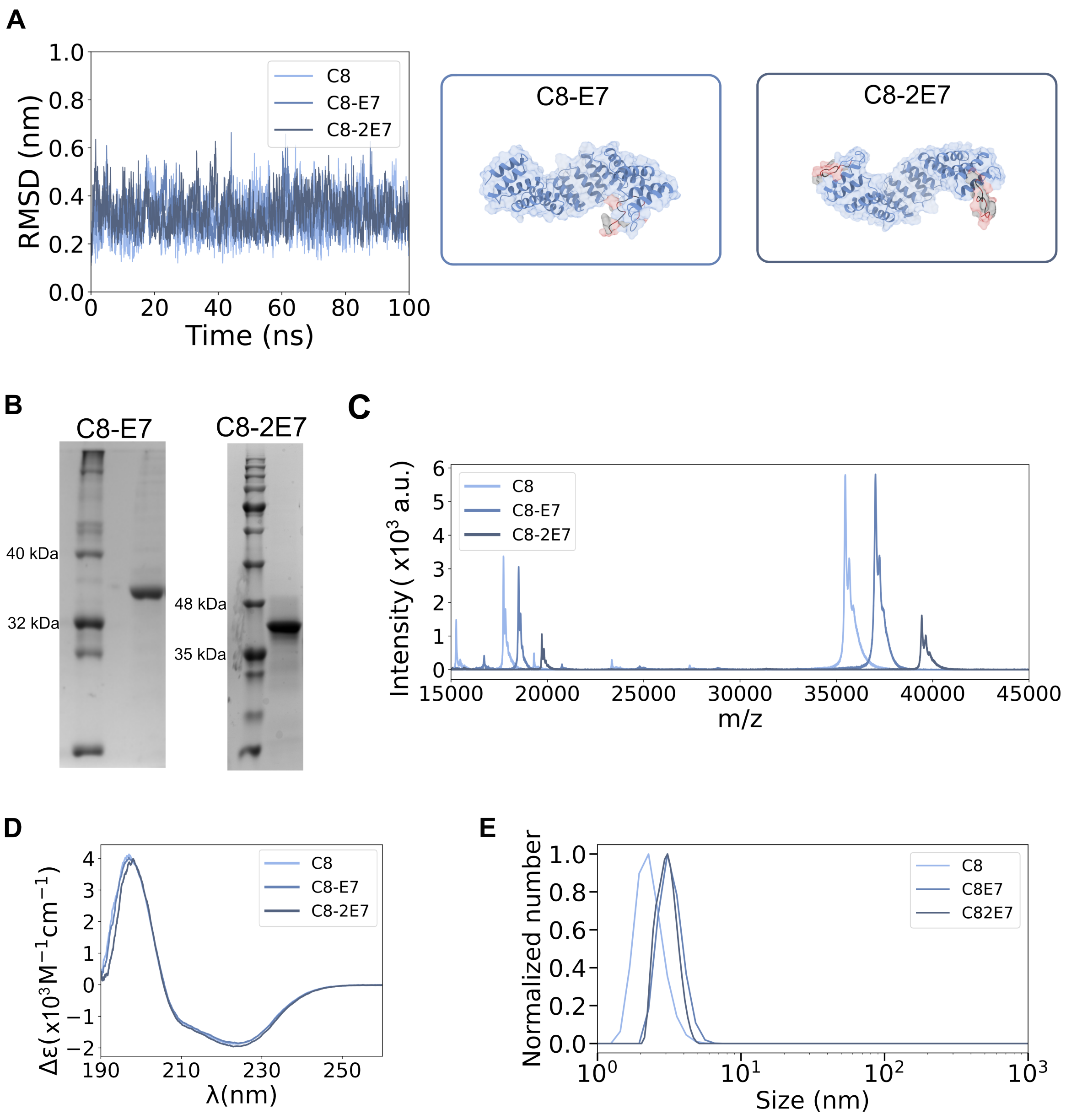

Figure S4. Characterization of the CTPR8 and the C8-E7 and C8-2E7 engineered proteins. **A.** Atomistic simulations of the proteins. RMSD during the 100 ns simulations of the three proteins (left). Snapshot of the last simulation frame of the two engineered proteins. The CTPR section is colored in blue, and the E7 peptides are colored displaying the glutamic acids in red and the phenylalanines in black (right). **B.** 12% SDS-PAGE gel electrophoresis of the **C8-E7** (left) and **C8-2E7** (right) engineered proteins. **C.** MALDI-TOF spectrum of the three proteins. The peak at 35.5 kDa corresponds to the CTPR8, the peak at 37 kDa corresponds to the **C8-E7**, and the peak at 39.4 kDa corresponds to the **C8-2E7**. **D.** CD spectra of the three proteins showing identical secondary structure. **E.** DLS histograms showing normalized number-based size distribution of the three proteins, with hydrodynamic radius (R_H) values of 2.27 nm for CTPR8, 3.07 nm for **C8-E7**, and 3.08 nm for **C8-2E7**.

Thermal characterization of E7 fibers, C8-E7 and C8-2E7.

Thermal measurements. The melting studies were performed in the same equipment as for the Circular Dichroism experiments described in the main paper (see Materials and Methods). The temperature was increased from 22°C to 95°C. The CD data was recorded at 200 nm for the fibers to analyze the destabilization of the π-stacking and at 220 nm for the proteins to analyze the decrease of the α-helix content. The concentration used for the fibers was 100 μM and for the proteins 5 μM. The unfolded fraction (F_u) was obtained by

$$F_u = \frac{\Delta\varepsilon - F}{UF - F}, \qquad \text{Equation S1}$$

being F (molar ellipticity where the protein is folded) the mean of the first 5 values and UF (molar ellipticity where the protein is unfolded) the mean of the last 5 values. The molar ellipticity (Δε) was calculated as described in the main paper (see Materials and Methods). The data was fitted with a smoothed spline fitted function with Python. The melting temperature (T_m) was obtained by subtracting the temperature value at which the F_u is 0.5 from the fitted function. The data represented for the **E7** fibers is the Δε instead of the F_u since the fiber does not lose completely the secondary structure, and therefore the ellipticity cannot be normalized by the UF.

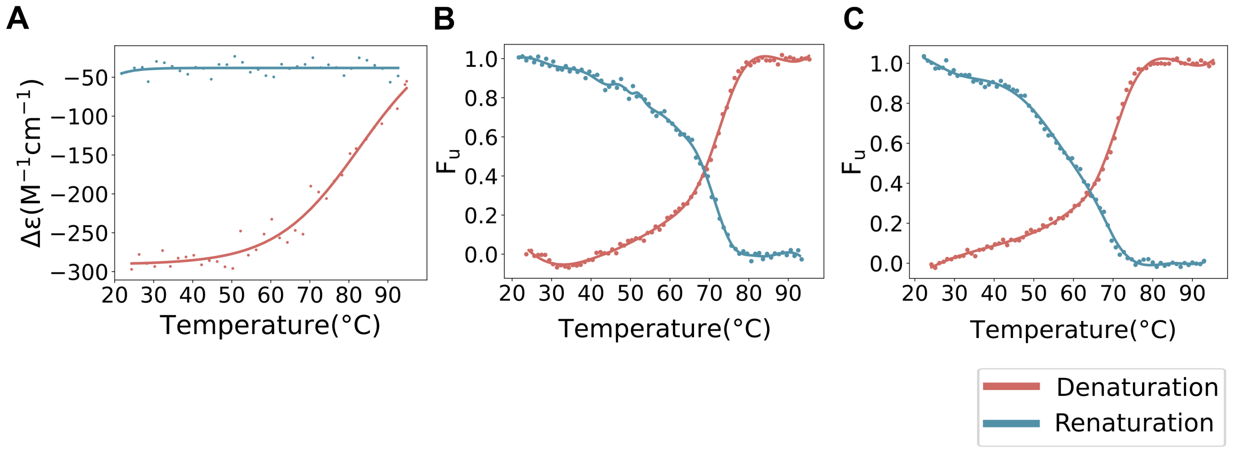

Figure S5. Thermal denaturation and renaturation curves. **A.** Δε of the **E7** fibers. **B.** F_u of **C8-E7**. **C.** F_u of **C8-2E7**.

Average diameter of the E7 fibers

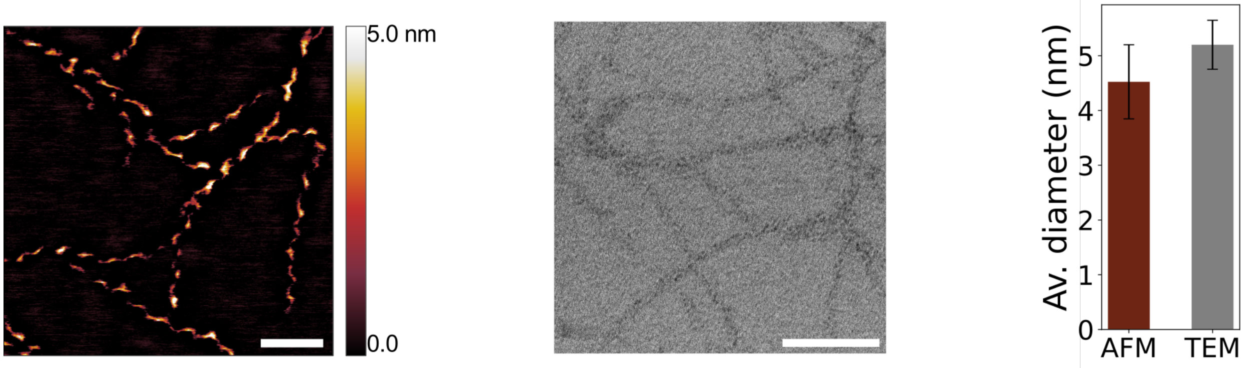

Figure S6. Characterization of the **E7** fiber. AFM (left), TEM (middle), and averaged diameter from the AFM and TEM images (right) of the **E7** fibers. Scale bars are 50 nm.

Characterization of the 2D coassembled material

Calculations performed for the estimation of the ratio between peptides and proteins. Considering that one protein is 3.8 nm high, that the distance between the peptides in the z-axis is 0.5 nm, and that there are two peptides facing each other to cover both sides of the fiber (Figure 4A):

$$\frac{1\ nm}{4\ peptides} = 0.25\ nm/peptide$$

$$\frac{3.8\ nm/protein}{0.25\ nm/peptide} = 15.2\ peptides/protein$$

Equation S2

Therefore, considering that the protein contributes itself 1 peptide, the ratio would be 1 protein per 14 peptides for a fiber decorated by just one side with the protein. For a double-sided decorated fiber, the amount of protein should be double:

$$\frac{2\ proteins}{14\ peptides} = \frac{1\ protein}{7\ peptides}$$

Equation S3

Thus, for a one-sided decorated fiber the ratio protein peptide is 1:14, whereas for a two-sided decorated fiber the ratio is 1:7.

Circular Dichroism. The measurements were performed at a concentration of 70.4 μM of **E7** fibers and 5 μM of **C8-2E7** (ratio 1:14). The methods of the measurements were the same as for the **C8-E7** and **C8-2E7** proteins in Figure S4D.

Atomic Force Microscopy (AFM). The equipment used was the same as described in the main paper (see Materials and Methods). For the measurements in Figure S8B the sample was deposited on top of a mica substrate following the same protocol as for the deposition and methodology for the self-assembled fibers. For the measurements in Figure S10A, B, and C the samples were deposited on top of mica substrate using the standard concentration of 1 mM fibers and 71 μM protein (dilution 1:1). For panel A the sample was deposited by drop-casting 10 μL of the coassembly, washing one time with 150 mM NaCl followed by a second wash of 50 mM $CaCl_2$. For panel B the sample was deposited and washed as in the panel A, followed by spin-coating. For panel C, 10 μL of the sample were directly deposited and immediately spin-coated. The characterization was performed on dry conditions using the Tapping mode. For the measurements in Figure S10D and E, 10 μL of the samples were deposited on top of a 300 nm SiO_2 substrate and the characterization was performed on dry conditions using the ScanAsyst mode. For panel D the coassembly was deposited at different dilutions (1:1, 1:10, and 1:100) both drop-casted and spin-coated. For panel E the fibers were deposited following the protocol described in the main paper, and the protein was directly drop-casted at 1 μM.

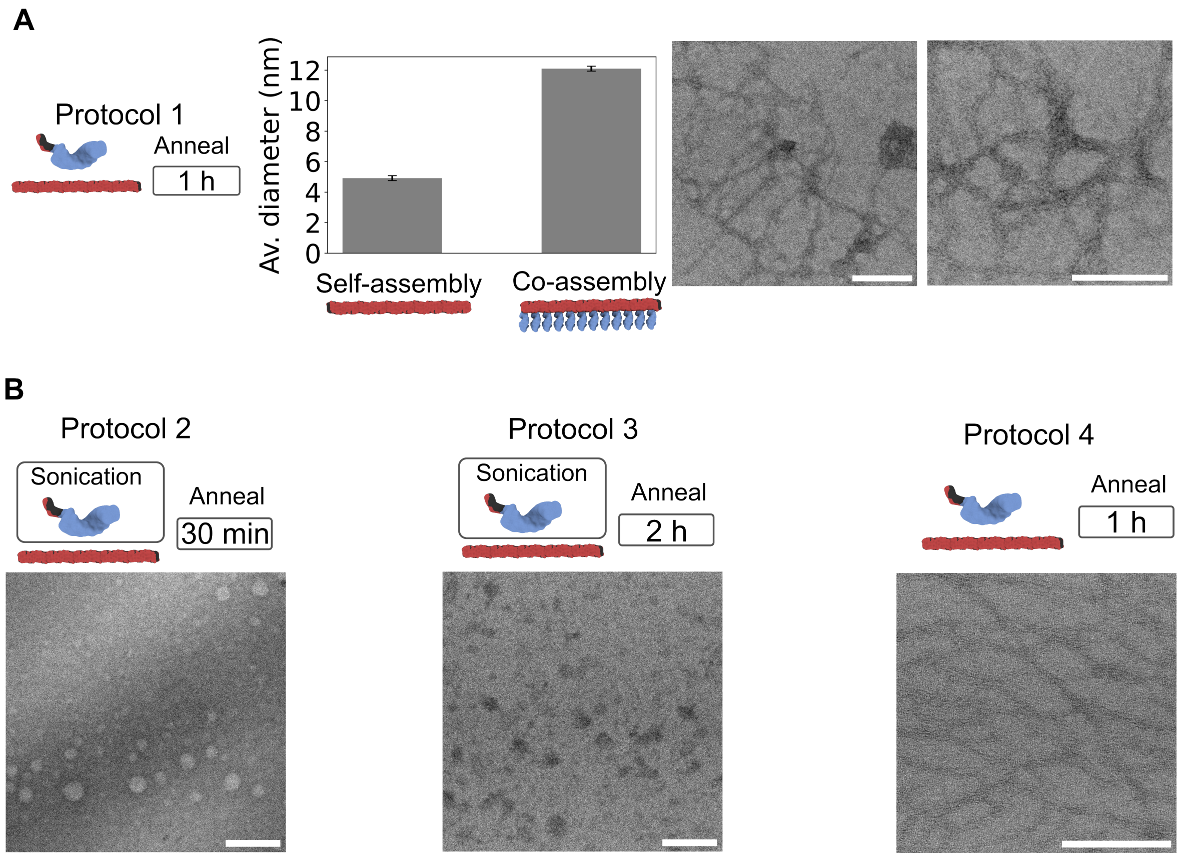

Figure S7. Protocol optimization for the coassembly. A. First protocol. The fibers and proteins are mixed annealed for 1 hour. The bar plot represents the average diameter of the two populations found in the sample. **B.** Additional protocols where the protein is sonicated before annealing (protocols 2 and 3) and annealed with the previously sonicated fibers for different times. The final protocol used for the coassembly is the protocol 4 in which the fibers are sonicated for 10 minutes, and the mixture is annealed for one hour. All scale bars are 100 nm.

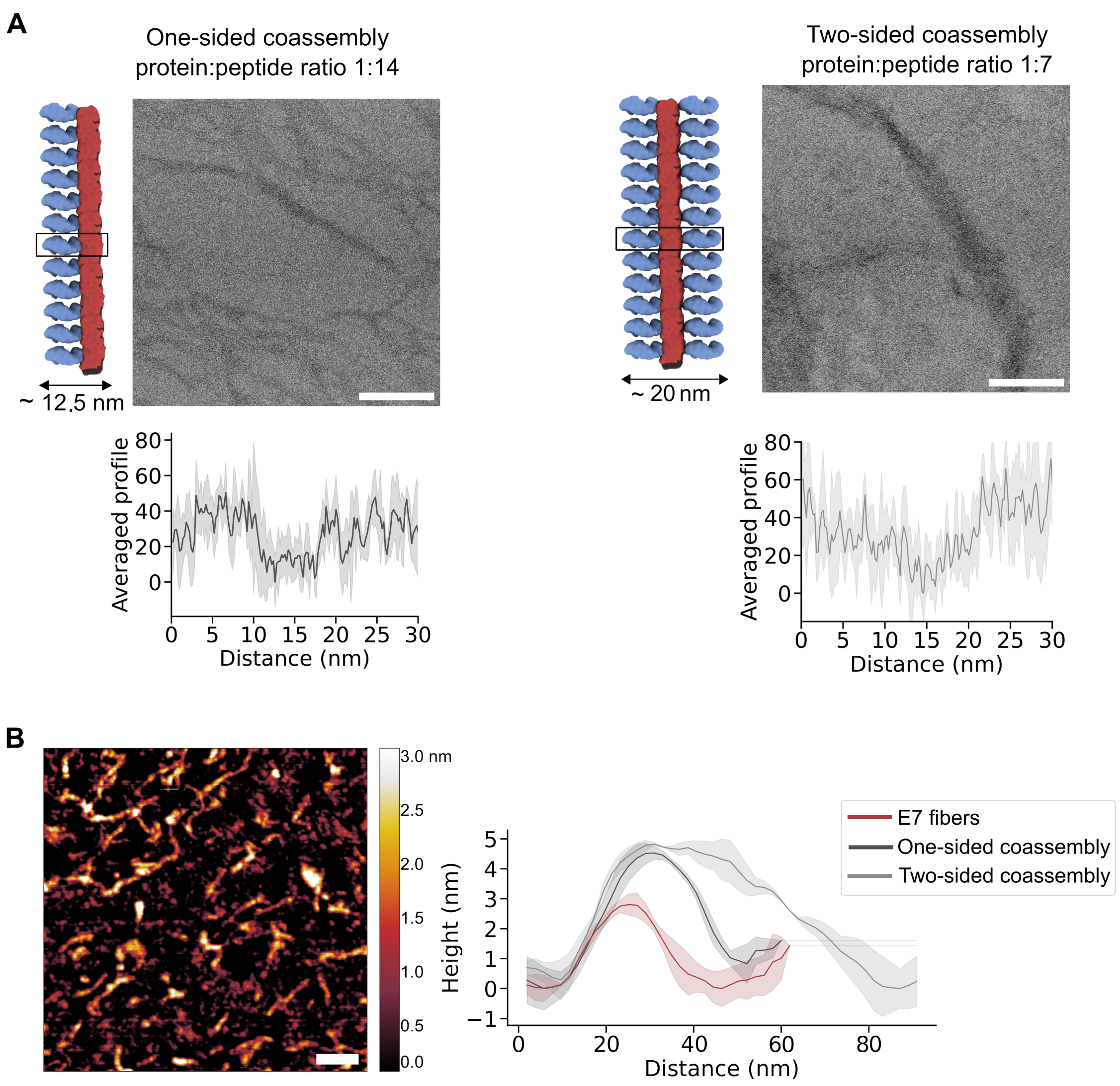

Figure S8. TEM and AFM characterization of the 1D coassemblies. A. TEM images of the 1D coassembled fibers with the 1:14 and the 1:7 protein:peptide ratios (top). Averaged profile from four different measurements of the TEM images on top and in Figure 4D. (bottom). **B.** AFM characterization of the 1D coassembled fibers with a 1:14 protein:peptide ratio. Height micrograph of the fibers (left). Averaged heights from different profiles for the three populations found in the sample, **E7** self-assembled fibers, one-sided, and two-sided coassemblies (right). All scale bars are 100 nm.

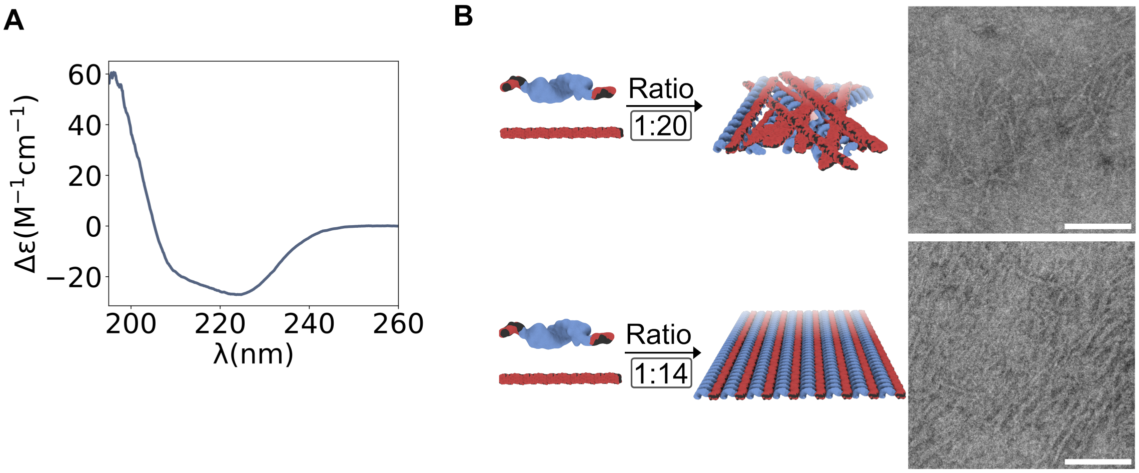

Figure S9. Validation of the protein stability within the assembly and optimal ratios. A. CD of the coassembly in which it can be appreciated the α-helical secondary structure of the **C8-2E7** protein. **B.** TEM images and graphical representations of the system containing **E7** fibers and the **C8-2E7** protein at 1:20 (excess of fiber) and 1:14 (coassembly) ratios. All scale bars are 100 nm.

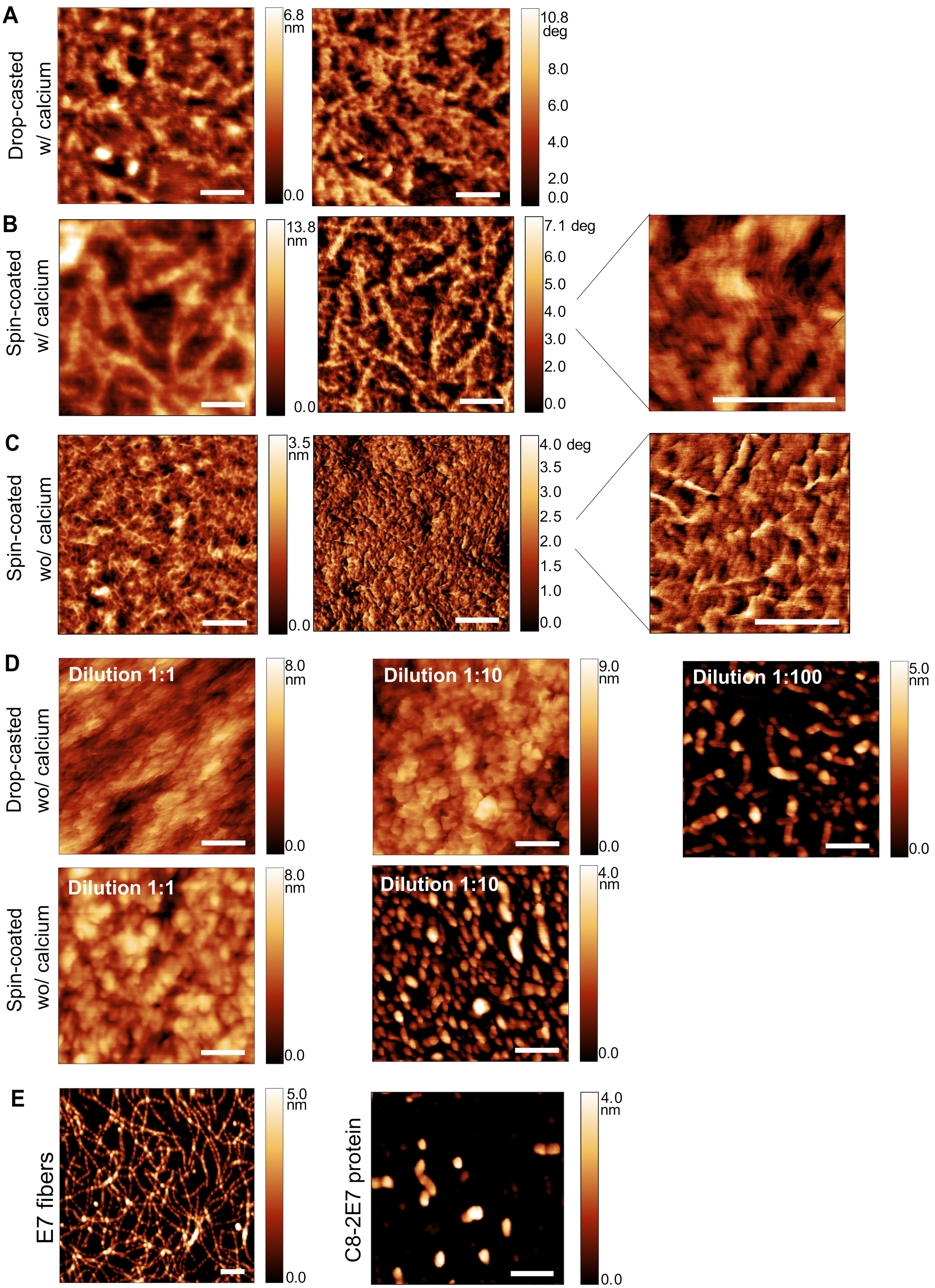
A
Drop-casted w/ calcium
6.8 nm
0.0
10.8 deg
8.0
6.0
4.0
2.0
0.0
B
Spin-coated w/ calcium
13.8 nm
0.0
7.1 deg
6.0
5.0
4.0
3.0
2.0
0.0
C
Spin-coated wo/ calcium
3.5 nm
0.0
4.0 deg
3.5
3.0
2.5
2.0
1.5
1.0
0.0
D
Drop-casted wo/ calcium
Dilution 1:1
8.0 nm
0.0
Dilution 1:10
9.0 nm
0.0
Dilution 1:100
5.0 nm
0.0
Spin-coated wo/ calcium
Dilution 1:1
8.0 nm
0.0
Dilution 1:10
4.0 nm
0.0
E
E7 fibers
5.0 nm
0.0
C8-2E7 protein
4.0 nm
0.0

Figure S10. AFM characterization of the 2D coassembly at varying substrates, dilutions and deposition methods. A, B, C. Characterization coassembly on top of mica substrate. Drop-casting (DC) with calcium (**A**). Spin-coated (SC) with calcium (**B**). SC w/o calcium, (**C**). **D.** Characterization of the coassembly on top of silica substrate. Different concentrations (dilution 1:1, 1:10, and 1:100) DC or SC. **E. E7** fibers and **C8-2E7** protein as controls on silica substrate. All sale bars are 100 nm.

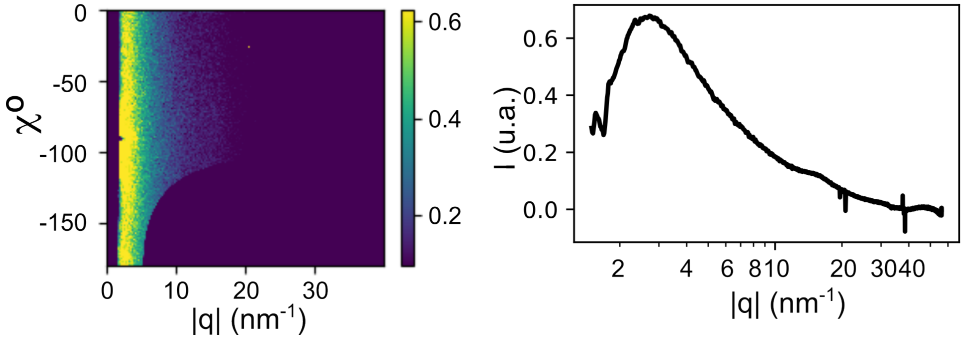

Figure S11. GIWAXS characterization of the disassembled 2D material at a 1:100 dilution. Scattering patterns at different azimuth angles (χ) (left). 2D intensity profile of the scattering patterns (right).

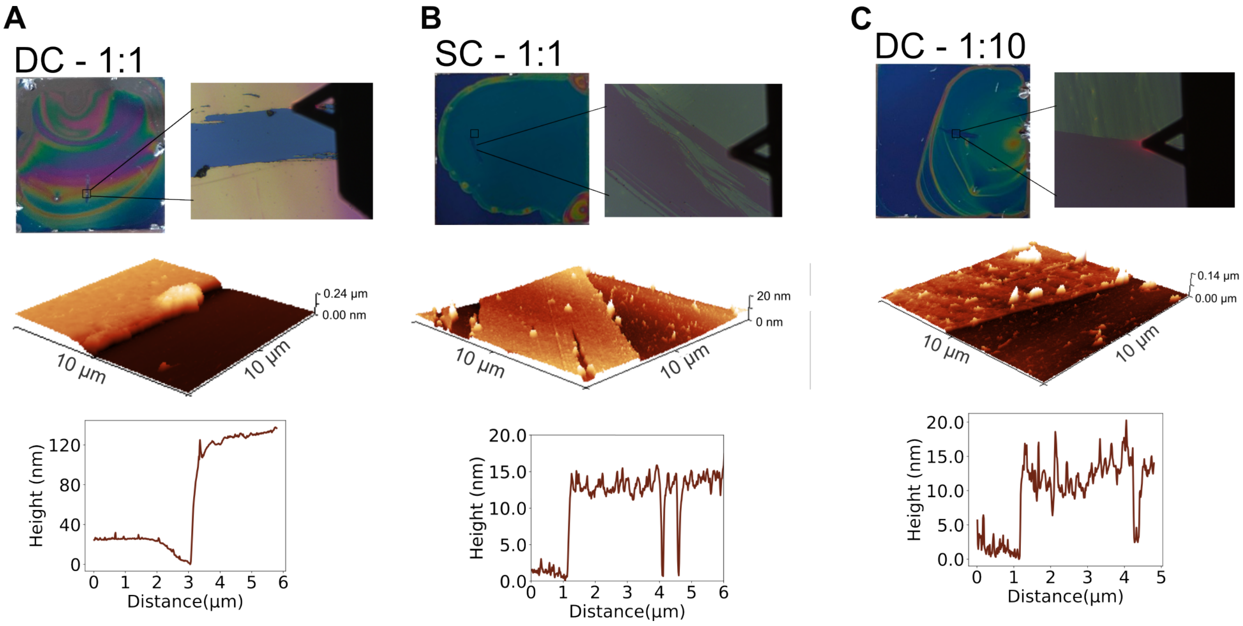

Figure S12. Thickness of the 2D coassembled films. A. Drop-casted (DC) at a dilution 1:1. **B.** DC at a dilution 1:10. **C.** SC at a dilution 1:1. In all the panels: Microscopy images of the films on top of a SiO_2 substrate (top). AFM characterization (middle and down). 3D image of the incision made in the films (middle), and the height profile (down).

Ionic conductivity in 2D coassemblies

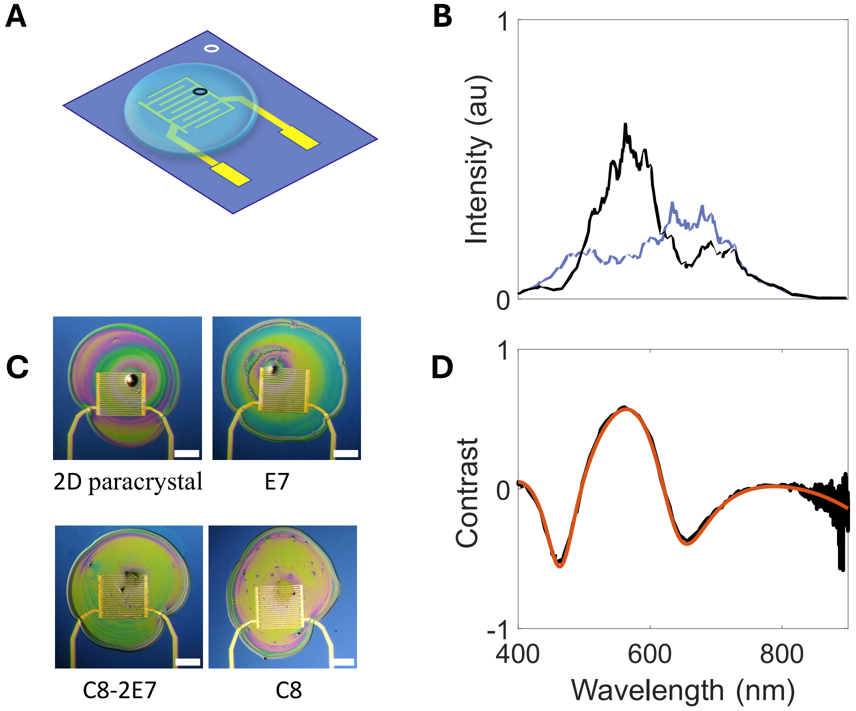

Figure S13. A. Diagram of the experimental device, indicating the locations where the micro-reflectance spectra were acquired at the films and the silicon oxide substrate, respectively. **B.** Micro-reflectance intensity spectra acquired at the bare substrate (purple curve) and the 2D paracrystal (black curve). **C.** Optical images of the 2D paracrystal, **E7** fibers, **C8-2E7** and CTPR8 protein film devices prepared by drop-casting. (Scale bar 0.5mm). **D.** Optical contrast derived from the data in panel B, with the red line representing a fit to a Fresnel model, yielding an approximate thickness of 250 nm for the 2D paracrystal.

Table S2. Thickness values for the **E7** fibers, CTPR8, **C8-2E7**, and 2D paracrystal films in Figure S13C, extracted from the analysis of micro-reflectance data. Thickness values are calculated as the average of results from measurements at four different locations at each sample. Error is estimated as the standard deviation.

	**Thickness (nm)**
**E7**	90 ± 20
CTPR8	100 ± 10
**C8-2E7**	80 ± 10
2D paracrystal	280 ± 60

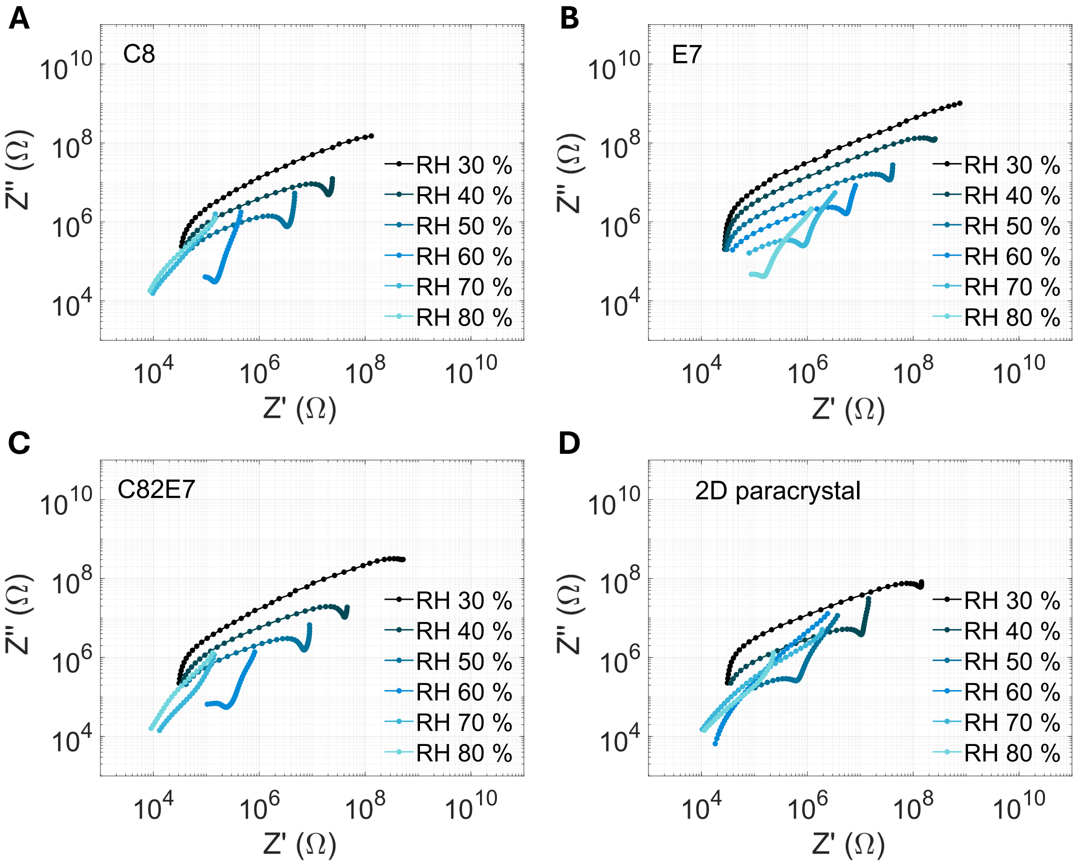

Figure S14. **Nyquist plots of impedance spectra for three different materials. A.** CTPR8 protein, **B. E7** fibers, **C. C8-2E7** and, **D.** 2D paracrystal at different relative humidity levels from 30% to 80% (logarithmic scale).

Table S3. Resistance values of **E7** fibers, CTPR8, **C8-2E7**, and 2D paracrystal films at different relative humidity levels ranging from 30% to 80%. All the resistances were obtained from the fitting of Nyquist plots to an elliptical function (see Methods), except those marked with *, for which the resistance was calculated by applying a linear fit to the diffusion tail and determining its intercept with the real impedance axis (Z') (see Methods and Figure S15).

**RESISTANCE (Ω)**						
R.H. (%)	30	40	50	60	70	80
**E7**	2.0E+09	3.2E+08	3.3E+07	4.9E+06	8.2E+05	1.5E+05
CTPR8	3.0E+08	1.9E+07	2.9E+06	1.4E+05	*5.6E+03	*5.7E+03
**C8-2E7**	7.0E+08	4.5E+07	6.8E+06	3.1E+05	*7.1E+03	*4.5E+03
2D paracrystal	1.5E+08	1.5E+07	7.9E+05	1.8E+04	*5.4E+03	*3.8E+03

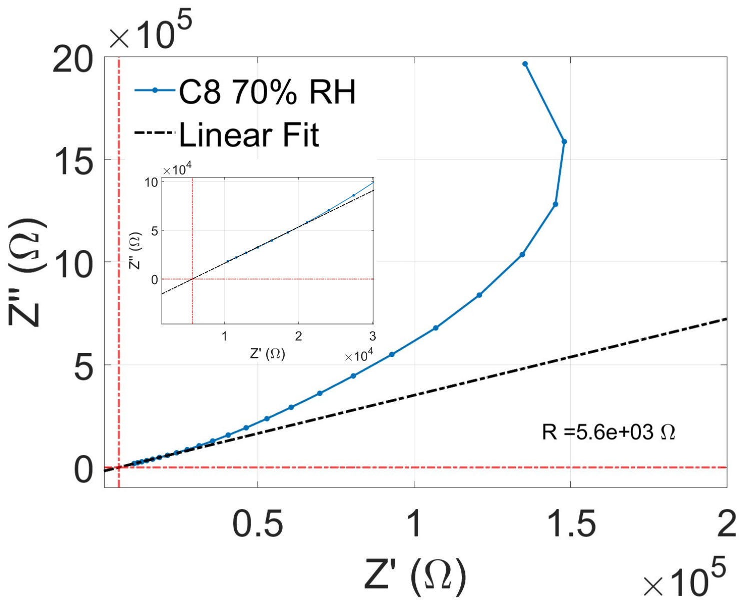

Figure S15. Nyquist plot of the impedance data for the CTPR8 protein at 70% relative humidity (RH) (blue curve). The resistance was estimated by applying a linear regression (black dashed line) to the diffusion tail and identifying its intersection with the real impedance axis (Z'), indicated by the cutoff between the two red dashed lines. The estimated resistance value is approximately 5.6 kΩ. Data points marked with (*) in Table S3 were calculated using this method. The inset shows a zoomed-in of the data.

Table S4. Conductivity values of **E7** fibers, CTPR8, **C8-2E7**, and 2D paracrystal films at different relative humidity levels ranging from 30% to 80% calculated from the resistance values in Table S3 and the corresponding thickness in Table S2.

**Conductivity (S/cm)**						
R.H. (%)	30	40	50	60	70	80
**E7**	6.77E-8	4.27E-7	4.18E-6	2.78E-5	1.65E-4	9.08E-4
CTPR8	4.02E-7	6.38E-6	4.24E-5	8.75E-4	*2.19E-2	*2.14E-2
**C8-2E7**	1.75E-7	2.74E-6	1.80E-5	3.95E-4	*1.73E-2	*2.73E-2
2D paracrystal	3.69E-7	2.98E-6	5.5E-5	2.5E-3	*8.0E-3	*1.14E-2

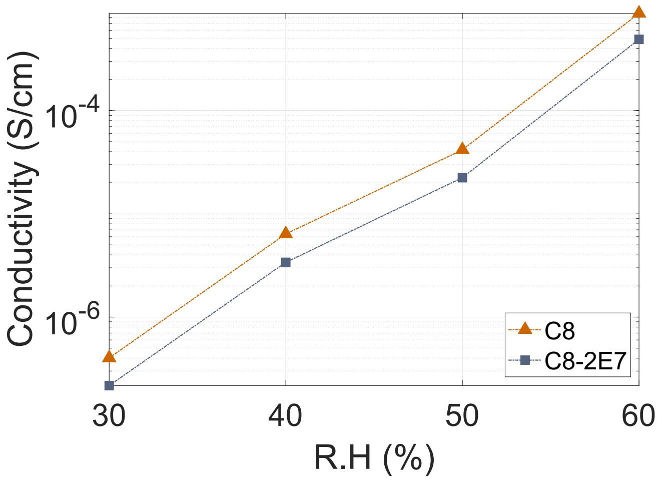

Figure S16. Conductivity as a function of relative humidity, ranging from 30% to 60%, for films of the **C8 -2E7**, and the CTPR8 protein, respectively.
